# Gelatin/Cerium-Doped Bioactive Glass Composites for Enhancing Cellular Functions of Human Mesenchymal Stem Cells (hBMSCs)

**DOI:** 10.3390/gels11060425

**Published:** 2025-06-01

**Authors:** Andrey Iodchik, Gigliola Lusvardi, Alfonso Zambon, Poh Soo Lee, Hans-Peter Wiesmann, Anne Bernhardt, Vera Hintze

**Affiliations:** 1 Institute of Materials Science, Max Bergmann Center of Biomaterials, TU Dresden University of Technology, Budapester Str. 27, D-01069 Dresden, Germany; andrey.iodchik@tu-dresden.de (A.I.); poh_soo.lee2@tu-dresden.de (P.S.L.); hans-peter.wiesmann@tu-dresden.de (H.-P.W.); 2 Department of Chemical and Geological Sciences, University of Modena and Reggio Emilia, 41125 Modena, Italy; gigliola.lusvardi@unimore.it (G.L.); alfonso.zambon@unimore.it (A.Z.); 3 Centre for Translational Bone, Joint and Soft Tissue Research, University Hospital “Carl Gustav Carus”, TU Dresden University of Technology, Fetscherstraße 74, D-01307 Dresden, Germany; anne.bernhardt@tu-dresden.de

**Keywords:** hydrogels, methacrylated gelatin, cerium-doped mesoporous bioactive glasses, human mesenchymal stem cells, osteogenic differentiation

## Abstract

Delayed or non-healing of bone defects in an aging, multi-morbid population is still a medical challenge. Current replacement materials, like autografts, are limited. Thus, artificial substitutes from biodegradable polymers and bioactive glasses (BGs) are promising alternatives. Here, novel cerium-doped mesoporous BG microparticles (Ce-MBGs) with different cerium content were included in photocrosslinkable, methacrylated gelatin (GelMA) for promoting cellular functions of human mesenchymal stem cells (hBMSCs). The composites were studied for intrinsic morphology and Ce-MBGs distribution by scanning electron microscopy (SEM) and energy-dispersive X-ray spectroscopy (EDX). They were gravimetrically analyzed for swelling and stability, compressive modulus via Microsquisher^®^ and bioactivity by Fluitest^®^ calcium assay and inductively coupled plasma-optical emission spectrometry (ICP-OES), also determining silicon and cerium ion release. Finally, seeding, proliferation, and differentiation of hBMSCs was investigated. Ce-MBGs were evenly distributed within composites. The latter displayed a concentration-dependent but cerium-independent decrease in swelling, while mechanical properties were comparable. A MBG type-dependent bioactivity was shown, while an enhanced osteogenic differentiation of hBMSCs was achieved for Ce-MBG-composites and related to different ion release profiles. These findings show their strong potential in promoting bone regeneration. Still, future work is required, e.g., analyzing the expression of osteogenic genes, providing further evidence for the composites’ osteogenic effect.

## 1. Introduction

Delayed or non-healing of bone defects and fractures in an aging population with many multi-morbid patients is a serious burden for patients’ mobility and quality of life as well as for the economy of the health care system [1]. Current bone replacement materials, e.g., auto-, allo-, and xenografts, have restrictions due to limited availability, donor sited morbidity, immune rejection, and infection risk [2]. Artificial bone graft substitutes made of biodegradable polymers and bioactive glasses (BGs) are considered as potential alternatives [3]. Hydrogels of gelatin methacrylate (GelMA) are an inexpensive, photocrosslinkable material, tunable in its physicochemical properties [4]. GelMA was successfully applied for the osteogenic differentiation of mesenchymal stem cells (MSCs) in osteogenic medium [4,5]. Further, GelMA showed enhanced osteogenesis in a critical-size rat calvaria bone defect when combined with stem cells [5]. Still, with respect to bone applications, hydrogels display rather low bioactivity and poor mechanical properties [3,6]. Bioactivity is defined here as a material property to form bone-like hydroxylapatit (HAP) on its surface when immersed in serum-like solution [7]. BGs on the other hand are biocompatible, biodegradable, osteoconductive, and bioactive, forming bone-like HAP in contact with body fluids and inducing bone bonding of the implant [6,8]. In addition, a large number of studies have demonstrated that silicate-based BGs and their ionic dissolution products (IDPs) induce osteoblast differentiation and bone formation, stimulate in vitro—and in vivo—angiogenesis and display antibacterial as well as anti-inflammatory properties, thus being highly attractive for bone tissue engineering (TE) applications [9]. The incorporation of ions into the silicate network, such as cerium, strontium, silver, and zinc, and their subsequent release is of high potential for further enhancing, e.g., osteogenesis and angiogenesis, antibacterial activity, and immunomodulation [9,10,11]. In the context of bone regeneration, cerium-doped BG particles might be an interesting component to be considered. Cerium oxide nanoparticles, e.g., were shown to act as multi-enzyme mimetics or radical scavengers, e.g., dismutating or scavenging reactive oxygen species (ROS) [12]. Redox enzyme-like activities, e.g., catalase and superoxide dismutase, were also found for melt quenched as well as for sol–gel-derived mesoporous cerium-doped bioactive glass microparticles (Ce-MBGs) [13,14,15,16]. This is of importance in the context of the so-called surgical stress response when implanting biomaterials with the production of ROS promoting inflammation [14]. Applying biomaterials with antioxidant properties could foster recovery and diminish the amount of anti-inflammatory drugs given to patients [14]. Next to their antioxidant properties, cerium-doped BGs are well known for their antibacterial, osteogenic, and angiogenic properties [15]. Recently, Westhauser et al. showed that IDPs of cerium-doped BG nanoparticles (Ce-BGNs) promote the proliferation, osteogenic differentiation, and extracellular matrix formation of human bone marrow-derived mesenchymal stem cells (hBMSCs) in a positive concentration-dependent way [17]. Combining natural polymer-derived hydrogels and inorganic fillers like BG nano- and microparticles is a promising biomimetic approach that might result in highly biocompatible composites closer matching the mechanical requirements of the implantation site, synergistically stimulating the desired cellular response, while having well-defined degradation profiles and bioactive properties [6,18,19,20]. In this context, it has been recently demonstrated that Ce-BGN-incorporated alginate/gelatin scaffolds improved the compressive modulus, viability, as well as osteogenic differentiation of hBMSCs [21]. In another study, scaffolds composed of chitosan and hollow, mesoporous, Ce-containing BG microspheres promoted the proliferation of directly inoculated hBMSCs in a cerium concentration-dependent manner [22]. Further, the osteogenic differentiation was enhanced when cells were grown in extracts of the respective scaffolds at higher cerium concentrations. Importantly, when applied in a rat calvarial defect model, these scaffolds significantly promoted bone regeneration when compared to those containing cerium-free BG. Also, Ce-MBGs implemented in alginate-based beads conveyed increased proliferation and oxidative stress resistance but no improved osteogenic differentiation to the mouse pre-osteoblastic cell line MC3T3-E1 [14].

The aim of the present study was to create novel composites based on GelMA and Ce-MBGs of the same composition as previously described [14] and to derive a more suitable cellular microenvironment with favorable physicochemical properties, promoting growth and osteogenic differentiation of hBMSCs. Here, the respective impact of MBG composition and concentration in composite materials on these properties were of special interest and also achieving homogeneous distribution in the composites. In this context, the derived composites were characterized with respect to swelling, elasticity, surface morphology, and spatial distribution of MBG particles as well as stability in cell culture medium (CCM). The acellular bioactivity of the composites (calcium depositions in CCM), including ion release profiles, was also studied. Further, the cellular attachment, proliferation, and osteogenic differentiation (ALP activity) of hBMSCs was assessed. The outcome of this study was expected to clarify which GelMA/Ce-MBG combination is best suited for promoting hBMSC functions and to serve as a promising base for further combinatory approaches promoting osteogenesis, e.g., loading with osteogenic drugs or modulating ion release and cellular response by additional electrical stimulation, and for their prospective application as bone replacement material in vivo.

## 2. Results and Discussion

### 2.1. Results

#### 2.1.1. Intrinsic Morphology of Composites and Distribution of MBGs

**Figure 1:**
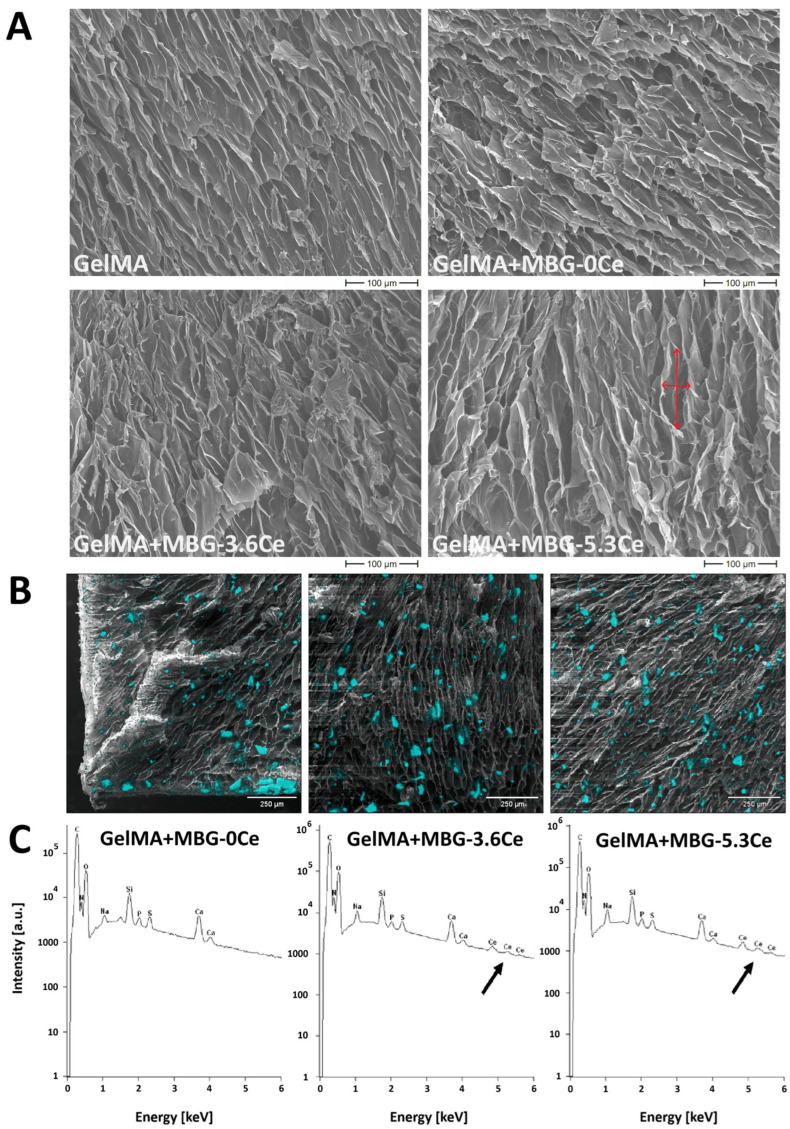
Intrinsic morphology and MBG distribution in composites with 1% *w*/*v* Ce-free and Ce-MBGs. (**A**) SEM images of composites (cross-section) containing 1% *w*/*v* of MBGs in comparison to pure GelMA. Short and long axis are highlighted with red arrows in the cross-section of GelMA + MBG-5.3Ce composite for illustration. Scale bar: 100 µm. (**B**) EDX-based silicon mapping and (**C**) elemental analysis of composites containing 1% *w*/*v* of different MBGs. Silicon location is superimposed on the images and depicted in cyan color; scale bar: 250 µm. Characteristic Ce peaks are highlighted with a black arrow.

**Figure A1:**
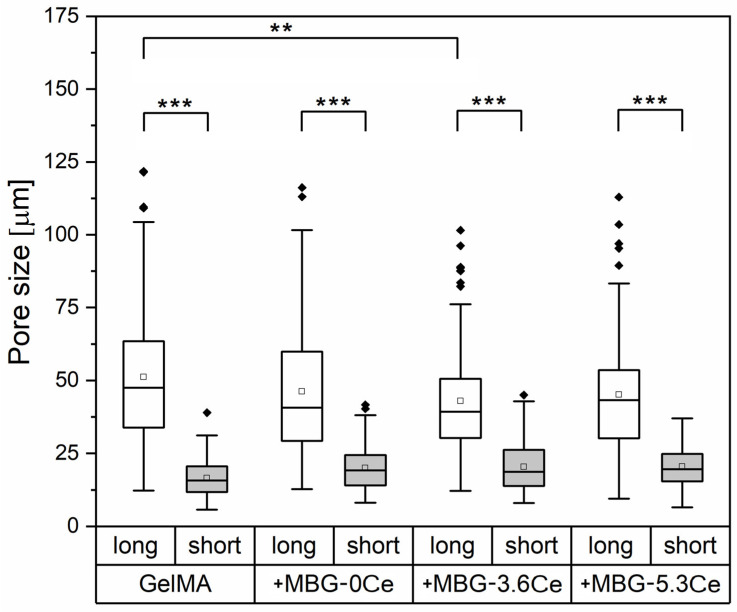
Grouped quartile bar chart representing pore size dispersion for long (red) and short (blue) axes for composites containing 1% MBGs in comparison to pure GelMA. The boxes indicate the first (Q1) and third quartiles (Q3), with the horizontal line inside the box showing the median (Q2) and the small square representing the mean. Whiskers extend to the smallest and largest values within 1.5 times the interquartile range (IQR) from Q1 and Q3. Black diamonds denote outliers beyond this range. Asterisks indicate statistical significance (two-way ANOVA), *** (*p* < 0.001) and ** (*p* < 0.01), with horizontal lines showing specific significant comparisons; *n* = 100 for each composite.

Cross-sections of the freeze-dried hydrogels were analyzed by SEM in order to estimate pore distribution and pore sizes of the scaffolds in the volume. When analyzing composites containing 1% *w*/*v* of different Ce-MBGs in comparison to MBG-0Ce and pure GelMA, an integrated and a highly porous structure (Figure 1A) with elongated pores orienting from top to bottom of the gels was observed with no major differences between samples. The quantitative analysis of the pore sizes revealed a significant difference between long and short axis of the pores for all composite types, proving their elongated shape (Figure A1). While there was no significant difference for the median value of the short axes (16–19.5 µm), this was the case for the long axes of GelMA and GelMA + MBG-3.6Ce composites (47.5 vs. 39.5 µm).

**Figure A2:**
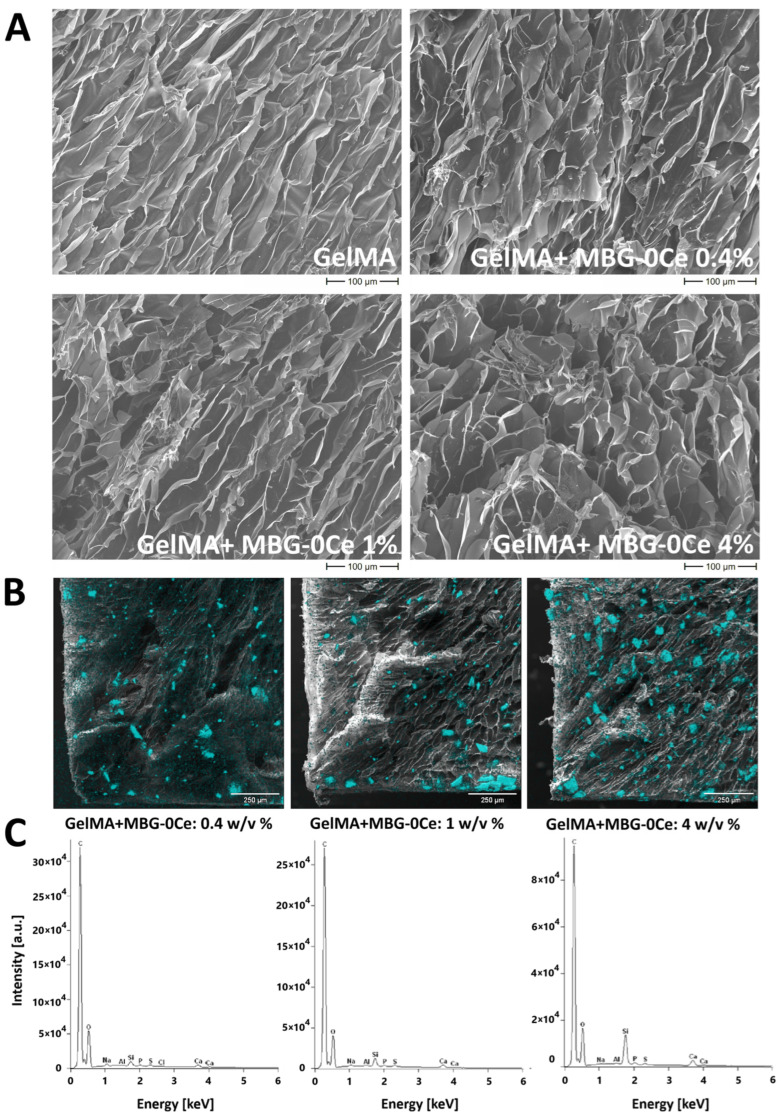
Intrinsic morphology and MBG distribution in composites with different concentrations of Ce-free MBGs. (**A**) SEM images of GelMA composites containing different concentrations of MBG in comparison to pure GelMA; scale bar: 100 µm. (**B**,**C**) Elemental analysis and silicon mapping of the same composites. Si location is superimposed on the images and depicted in cyan color; scale bar: 500 µm.

For composites containing different concentrations of MBG-0Ce (0.4, 1 and 4% *w*/*v*), there was a tendency of the pores to change their shape with increasing MBG content from elongated to a more irregular spherical when compared to pure GelMA (Figure A2). Further, the homogeneous distribution of MBGs, as a prerequisite for homogeneity in mechanical properties and a further application of the composites, was investigated by elemental analysis (EDX) and silicon mapping for each MBG-containing composite analyzing cross-sections (Figure 1 and Figure A2B,C).

**Figure A3:**
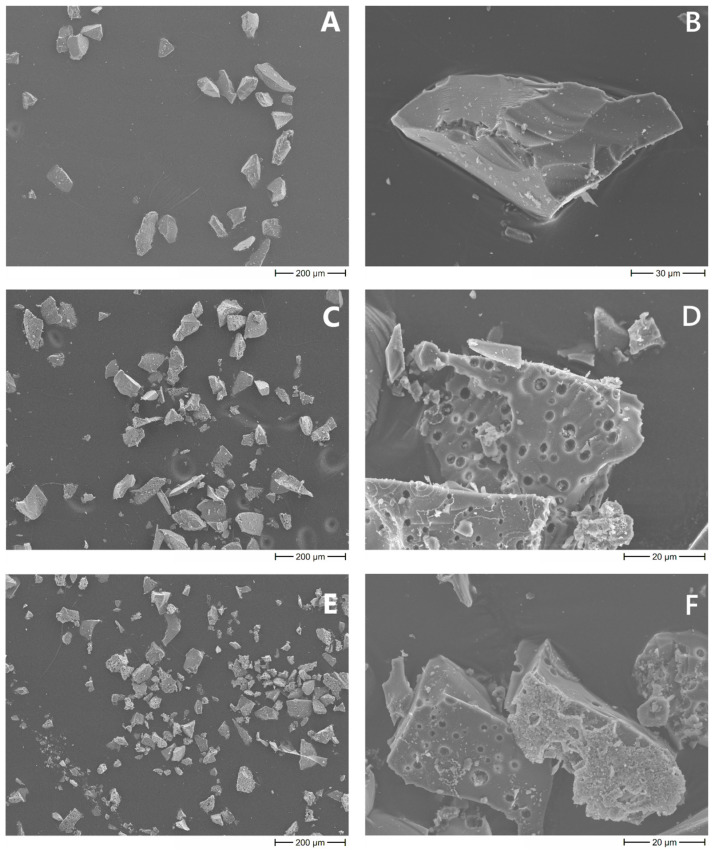
SEM images of MBG particles. (**A**,**B**) MBG-0Ce (scale bars: 200 µm and 30 µm); (**C**,**D**) MBG-3.6Ce and (**E**,**F**) MBG-5.3Ce (scale bars: 200 µm and 20 µm, both).

When analyzing composites containing 1% *w*/*v* of different Ce-MBGs in comparison to MBG-0Ce and pure GelMA, the MBGs were rather homogeneously distributed in the cross-section for all composites, with a slight tendency towards aggregation to the bottom of the hydrogel for MBG-0Ce (Figure 1B). The size of the particles colored by silicon mapping were generally below 100 µm in size, indicating no aggregation. As for SEM analysis of bare MBGs, the sizes were found to be in the range of 83 ± 25 µm, 73 ± 25 µm, and 49 ± 15 µm for the long axis of MBG-0Ce, MBG-3.6Ce, and MBG-5.3Ce and 39 ± 9 µm, 43 ± 10 µm, and 29 ± 9 µm for the short axis (Figure A3).

Elemental analysis showed characteristic bands of silicon and calcium elements in all composites (Figure 1C). For GelMA + MBG-0Ce, there was expectedly no cerium detected, while this was the case for GelMA + MBG-3.6Ce and GelMA + MBG-5.3Ce. When comparing the silicon mapping for composites with different concentrations of MBG-0Ce (0.4, 1 and 4% *w*/*v*), elemental analysis again revealed silicon and calcium elements in all samples (Figure A2C). As expected, the highest value for the silicon content was reached with 4% *w*/*v* MBG-0Ce.

#### 2.1.2. Swelling and Mechanical Properties of Composites

**Figure 2:**
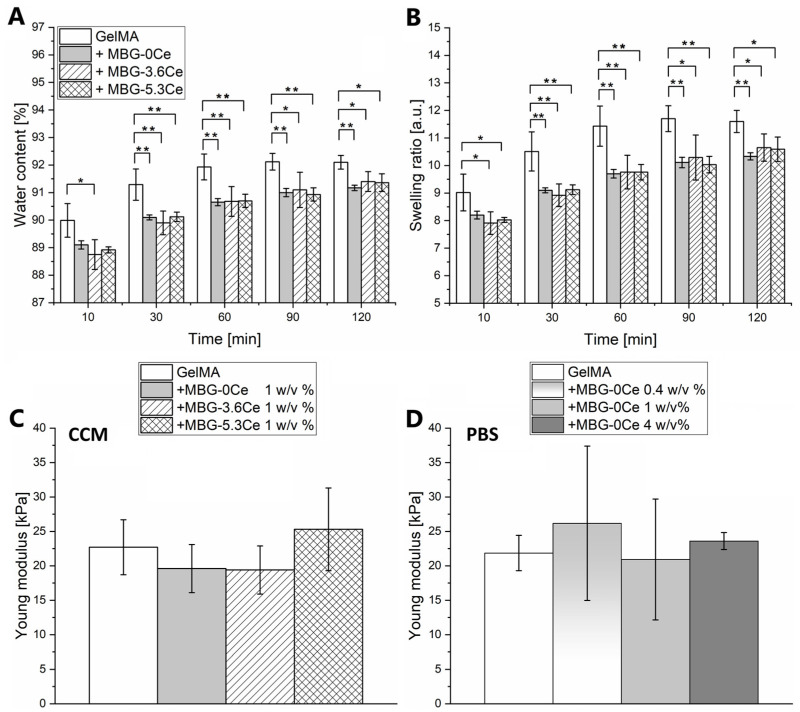
Swelling and mechanical properties. (**A**) Water content and (**B**) swelling ratio of composites containing 1% *w*/*v* of different MBGs in comparison to pure GelMA in CCM at 37 °C and 5% CO_2_ from 10 to 120 min (*n* = 4). One-way ANOVA: * *p* < 0.05, ** *p* < 0.01. (**C**) Young’s moduli of the same composites incubated at 37 °C and 5% CO_2_ in CCM (*n* = 3). (**D**) Young’s moduli of composites with different concentrations of MBG-0Ce incubated at 37 °C in PBS (*n* = 3). One-way ANOVA: no statistical differences.

**Figure A4:**
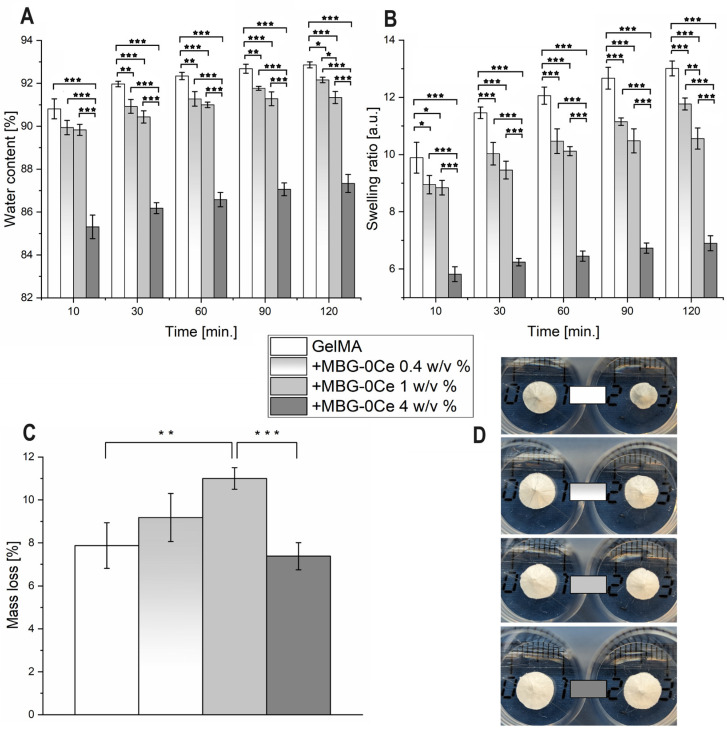
Swelling properties and stability of GelMA composites with different concentrations of Ce-free MBGs. (**A**) Water content and (**B**) swelling ratio of the composites with different concentrations of MBGs in PBS at 37 °C from 10 to 120 min (*n* = 4). (**C**) Mass loss of the hydrogels over 7 days in PBS at 37 °C (*n* = 4). (**D**) Images of the freeze-dried composites before (left) and after (right) incubation in PBS for 7 days. Standard deviation is depicted for each bar (*n* = 4). One-way ANOVA: * *p* < 0.05, ** *p* < 0.01, *** *p* < 0.001.

Swelling properties in cell culture medium (CCM) were assessed by gravimetrical measurements for composites containing 1% *w*/*v* MBG-0Ce in comparison to MBG-3.6Ce, MBG-5.3Ce, and pure GelMA (Figure 2A,B). All composites reached an equilibrium level of water content (*WC*) and swelling ratio (*SR*) after 90 to 120 min of the experiment. The addition of MBGs led to significantly decreased *WC* and *SR*, irrespective of MBG type, which was already apparent after 10 min swelling time. At 120 min swelling time, pure GelMA reached the highest *WC* of 92.1 ± 0.25% and *SR* of 11.6 ± 0.4, while with the addition of different MBG particles, both *WC* and *SR* decreased to about 91% and 10%, respectively. There was no significant impact of cerium content (Figure 2A,B). Further, the swelling properties were assessed in PBS at 37 °C for composites containing 0.4, 1, and 4% *w*/*v* MBG-0Ce (Figure A4A,B).

Again, all composites reached equilibrium level of *WC* and *SR* after 90 to 120 min. The addition of MBGs led to significantly decreased *WC* and *SR* depending on MBG-0Ce concentration, which was already apparent after 10 min swelling time. At 120 min swelling time, pure GelMA reached the highest *WC* of 92.86 ± 0.14% and *SR* of 13.01 ± 0.26, while composites containing 4% MBG-0Ce displayed the lowest values (*WC* of 87.34 ± 0.42%, *SR* 6.9 ± 0.26). Of note, no major differences in swelling behavior between PBS and CCM could be detected when comparing *SR* and *WC* of GelMA and GelMA + MBG-0Ce in both (Figure 2 and Figure A4A,B).

**Figure A5:**
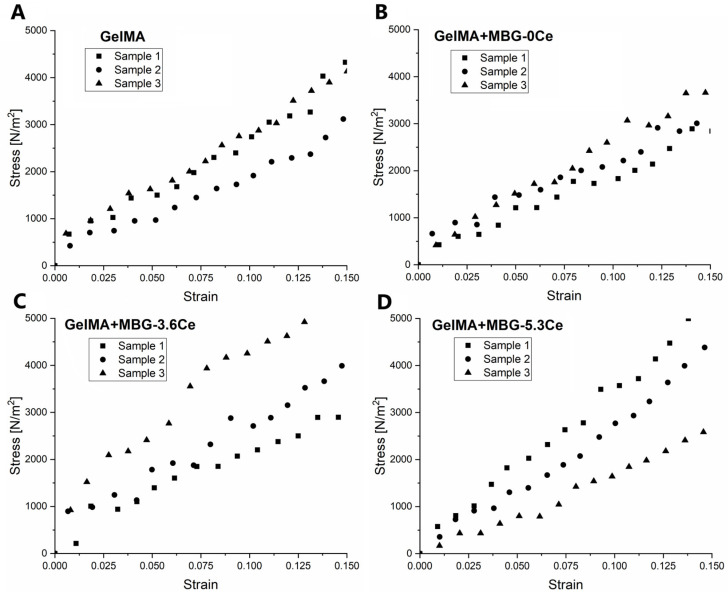
Stress–strain curves of composites containing 1% *w*/*v* of different MBGs. (**A**) GelMA; (**B**) GelMA + MBG-0Ce; (**C**) GelMA + MBG-3.6Ce; (**D**) GelMA + MBG-5.3Ce. Analysis was performed in comparison to pure GelMA in CCM at 37 °C (*n* = 3).

**Figure A6:**
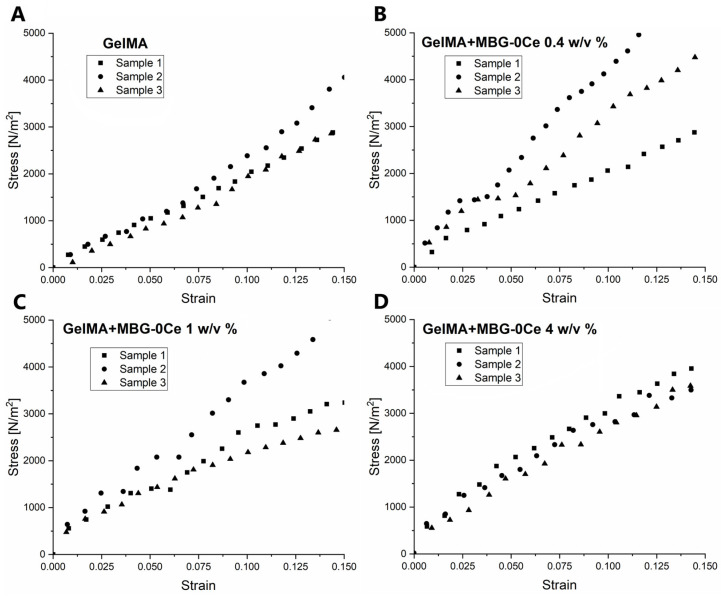
Stress–strain curves of composites with different concentrations of Ce-free MBGs. (**A**) GelMA, (**B**) GelMA + MBG-0Ce 0.4 *w/v %*; (**C**) GelMA + MBG-0Ce 1 *w/v %*; (**D**) GelMA + MBG-0Ce 4 *w/v %*. Analysis was performed in comparison to pure GelMA in PBS at 37 °C (*n* = 3).

The elastic modulus of the composites was determined by compressive loading and calculating the slope of the initial linear region (0–15%) of the strain–stress curves. Next, the mechanical properties were assessed in CCM for composites containing 1% *w*/*v* MBG-0Ce in comparison to MBG-3.6Ce, MBG-5.3Ce, and pure GelMA (Figure 2C and Figure A5). All composites demonstrated elastic behavior. No significant differences between the Young’s moduli were detected with values between 19.6 ± 3.5 kPa (GelMA + MBG-0Ce) and 25.3 ± 6 kPa (GelMA + MBG-5.3Ce). The mechanical properties of composites containing 0.4, 1, and 4% *w*/*v* of MBG-0Ce were assessed in PBS at 37 °C (Figure 2D and Figure A6). Again, all investigated composites showed elastic behavior, while their Young’s moduli were not significantly different. Values were between 20.93 ± 8.78 kPa (GelMA + 1% MBG-0Ce) and 26.18 ± 11.21 kPa (GelMA + 0.4% MBG-0Ce). Of note, no major difference could be detected when comparing the Young’s moduli of GelMA and GelMA + MBG-0Ce pre-swollen in PBS or CCM.

#### 2.1.3. Stability and Bioactivity of Composites

**Figure 3:**
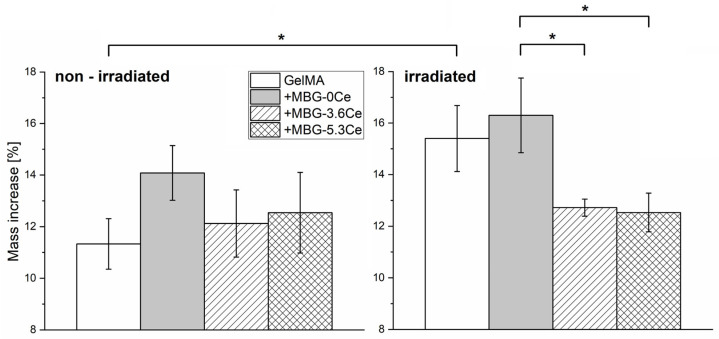
Stability of GelMA composites in CCM. Relative mass increase of composites containing 1% *w*/*v* of different MBGs in comparison to pure GelMA without gamma irradiation (**left**) and gamma-irradiated (**right**) after 28 days of incubation in CCM at 37 °C and 5% CO_2_ (*n* = 3). One-way ANOVA: * *p* < 0.05. Values between sterilized and non-sterilized gels were compared only for the same material type.

Assessing the stability of non-irradiated composites in CCM with regular medium changes at 37 °C and over 28 days revealed no significant differences between GelMA + MBG-0Ce in comparison to +MBG-3.6Ce, +MBG-5.3Ce, and pure GelMA (Figure 3). However, all the composites increased their mass with GelMA + MBG-0Ce showing the highest relative mass increase (14.08 ± 1.06%). This mass increase was also apparent when gamma-irradiating samples before stability assessment. Interestingly, there was a significant difference between GelMA + MBG0Ce and GelMA + MBG3.6Ce/5.3Ce composites detected (Figure 3).

The mass increase in gamma-irradiated GelMA + MBG-0Ce was 16.29 ± 1.46%, while for GelMA + MBG-3.6Ce and GelMA + MBG-5.3Ce, it was 12.72 ± 0.33% and 12.53 ± 0.75%, respectively. Further, there was a significant increase for GelMA after being irradiated (15.4 ± 1.28% vs. 11.33 ± 1.0%).

In addition, the stability of composites containing 0.4, 1, and 4% *w*/*v* of MBG-0Ce were assessed in PBS at 37 °C and over 7 days in comparison to pure GelMA. Here, a slight mass loss between 7 to 11% was observable for all samples (Figure A4C). Significant differences were only apparent between GelMA + 1% *w*/*v* MBG-0Ce and pure GelMA, as well as GelMA + 4% *w*/*v* MBG-0Ce. In line with this, with the exception of pure GelMA, the shape of the hydrogels was not strongly altered after 7 days in PBS. For GelMA, however, the samples increased in height but decreased in diameter (Figure A4D). The absolute mass increase in CCM after 28 days suggests the formation of precipitates on the composites that are derived from direct contact with CCM, which might include calcium phosphate deposition.

**Figure 4:**
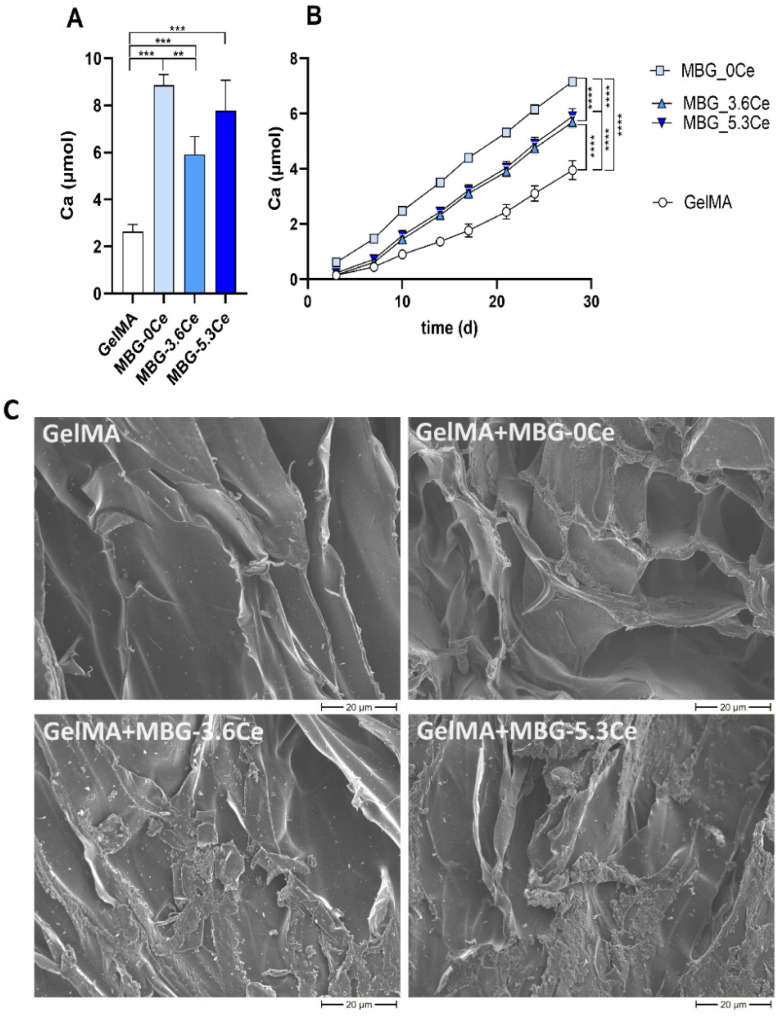
Bioactivity of composites. (**A**) Calcium content in the destroyed and HCl-treated gamma-irradiated composites containing 1% *w*/*v* of different MBGs in comparison to pure GelMA after 28 days of incubation in complete CCM (*n* = 3). One-way ANOVA: ** *p* < 0.01, *** *p* < 0.001. (**B**) Accumulated calcium uptake of the composites. Differences between cell culture medium before and after incubation with the composites (measured by ICP-OES) was accumulated for every medium change; *n* = 4. Two-way ANOVA, followed by Tukey’s multiple comparison test, with **** *p* < 0.0001. (**C**) SEM images of freeze-dried composites containing 1% *w*/*v* of different MBGs in comparison to pure GelMA after 28 days of incubation in CCM; scale bar: 20 µm.

To further confirm this, calcium content on gamma-irradiated composites incubated in CCM over 28 days with regular medium changes was quantified after final dissolution the constructs of in HCl (Figure 4A).

The following colorimetric analysis revealed high calcium content, with values of MBG-containing gels significantly exceeding those for GelMA. The highest values were observed for GelMA + MBG-0Ce and GelMA + MBG-5.3Ce, amounting to 8.9 µmol ± 0.5 and 7.8 ± 1.3 µmol, respectively, while for GelMA + MBG-3.6Ce, the calcium amount was 5.9 ± 0.75 µmol and for GelMA it was only 2.6 ± 0.3 µmol. Notably, there was no statistically significant difference between Ce-MBG-containing composites. It is worth mentioning that one has to consider the amount of calcium present in MBGs inside the composites before incubating in CCM. Bioactive glass dissolution is a complex phenomenon that includes both calcium release to the medium and uptake from it. However, if hypothetically assuming dissolution only at the end of the experiment, after treating the composites with HCl, one would obtain only 1.5–1.9 µmol, depending on MBG type, attributed only to calcium inside the particles. Thus, the present results suggest a high uptake from CCM during incubation. To estimate the uptake of calcium during the 28 days incubation of gamma-irradiated composites in CCM, the conditioned media were collected on each respective medium change day and analyzed via ICP-OES. The difference between the original calcium concentration in the medium and the quantified concentration was defined as calcium uptake. After 3 days of incubation, all composites had already began absorbing calcium from the medium, rather than releasing it, as could be expected for MBG-containing hydrogels (Figure 4B). As for the directly determined calcium deposition (Figure 4A), composites containing MBG-0Ce displayed the highest uptake of calcium, while it was lower for Ce-MBGs and lowest for pure GelMA (Figure 4B). The accumulated calcium uptake after 28 days of incubation, measured by ICP-OES, was 7.2 ± 0.1 µmol for GelMA + MBG-0Ce, 5.7 ± 0.2 µmol and 5.9 ± 0.3 µmol for GelMA + MBG-3.6Ce and GelMA + MBG-5.3Ce, respectively, as well as 4.0 ± 0.3 µmol for GelMA.

Finally, to visualize calcium deposition on composites, cross-sections were analyzed via SEM (Figure 4C). There was a marked difference between GelMA and composites containing 1% *w*/*v* MBGs after 28 days of incubation in CCM. While only the presence of single sub-micrometer-sized particles was observable for GelMA, the composites demonstrated significant plate-like precipitates occupying the entire biopolymeric walls of the scaffolds, likely attributing to hydroxyapatite formation, as reported previously [23]. However, this aspect needs to be confirmed by XRD/FTIR in future investigations.

#### 2.1.4. Cell Proliferation and Differentiation

**Figure 5:**
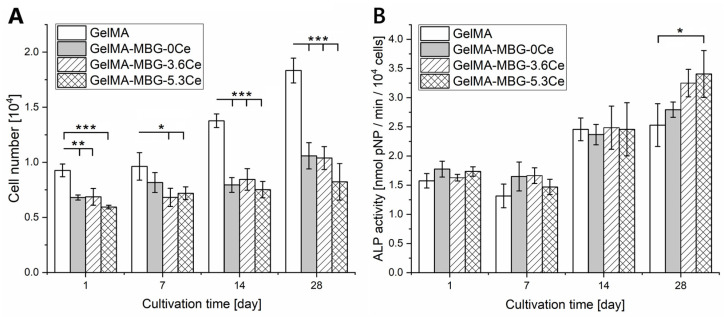
Cell proliferation and osteogenic differentiation. Cell number (LDH assay; (**A**)) and specific ALP activity (ALP assay; (**B**)) of hBMSCs cultivated on composites containing 1 *w*/*v* % of different MBGs in comparison to pure GelMA. One-way ANOVA: * *p* < 0.05; ** *p* < 0.01; *** *p* < 0.001; *n* = 4.

**Figure A7:**
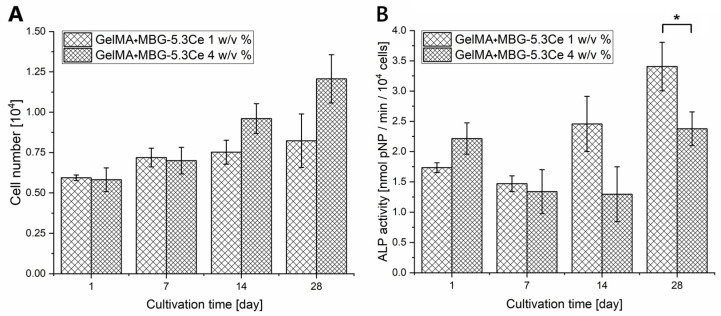
Cell number and specific ALP activity of hBMSCs cultivated on composites containing 1 *w*/*v* % or 4 *w*/*v* % MBG-5.3Ce; LDH (**A**) and ALP (**B**) assays; one-way ANOVA: * *p* < 0.05; *n* = 4.

The cellular proliferation and osteogenic differentiation of hBMSCs was investigated after directly seeding the cells on the composites. To increase seeding efficiency, seeding of composites was carried out in sterile Teflon rings. Motivated by their hydrophobic nature as well as the close contact of the swollen gels and the rings, it was anticipated that the initially seeded 20,000 hBMSCs would preferentially adhere to the surface of the composites. For GelMA, on day 1, about 9300 metabolically active cells could be found on the gels, which is 47% of the initially seeded cell number (Figure 5A). In the case of MBG-containing composites, the initially seeded cell number was about 6800, 6860, and 5940 for GelMA + MBG-0Ce, GelMA + MBG-3.6Ce, and GelMA + MBG-5.3Ce with 1 *w*/*v* % MBGs, respectively. For GelMA + MBG-5.3Ce 4 *w*/*v* %, it was 5810 (Figure A7A). This relates to about 30% to 34% of the initially seeded cell number. Overall, the presence of MBGs seemed to result in decreased cellular attachment in comparison with pure GelMA.

Cell proliferation over 28 days was significantly higher for pure GelMA in comparison to composites (Figure 5A). Cell number on GelMA samples increased about two times between day 1 and day 28, while it increased only 1.56-fold for GelMA + MBG-0Ce, 1.51-fold for GelMA + MBG-3.6Ce, and 1.39-fold for GelMA + MBG-5.3Ce with 1 *w*/*v* % MBGs. For GelMA + MBG-5.3Ce 4 *w*/*v* %, the increase in cell number was two-fold again (Figure A7A). Still, the presence of MBGs in the composite decreased proliferation in comparison to pure GelMA.

**Figure A8:**
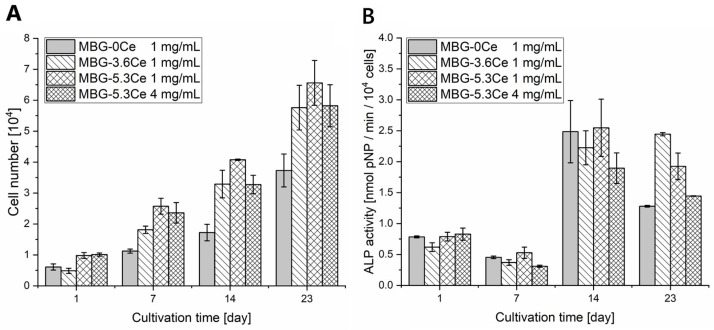
Cell proliferation and osteogenic differentiation. Cell number and specific ALP activity of hBMSCs cultivated in extracts from 1 mg/mL and 4 mg/mL of different MBGs: LDH (**A**) and ALP (**B**) assays. *n* = 2.

Interestingly, in indirect cell culture with extracts of bare MBGs, the effect of Ce-MBGs was different. Here, extracts from MBG-3.6Ce and MBG-5.3Ce increased hBMSC proliferation in contrast to cerium-free MBG-0Ce, which could not be further enhanced by a higher concentration of MBG-5.3Ce (Figure A8A).

hBMSCs already demonstrated elevated ALP levels on day 1 for pure GelMA and composites, hinting at their altered state after being seeded on the gels (Figure 5B). In contrast, when seeded on TCPS, ALP-levels were nearly three-fold lower. While specific ALP activity was similar for all composites and GelMA after 14 days of cultivation, it was elevated for all Ce-MBG containing composites compared to GelMA after 28 days of cultivation. This was significant for GelMA + MBG5.3Ce, suggesting a stimulating effect of Ce-MBGs on osteogenic differentiation of hBMSCs in particular at later time points. Interestingly, this effect could not be increased by 4 *w*/*v* % MBG5.3Ce (Figure A7B). Instead, it was significantly reduced compared to MBG5.3Ce 1 *w*/*v* %. This observation was supported in indirect cell culture with extracts of bare MBGs (Figure A8B).

**Figure 6:**
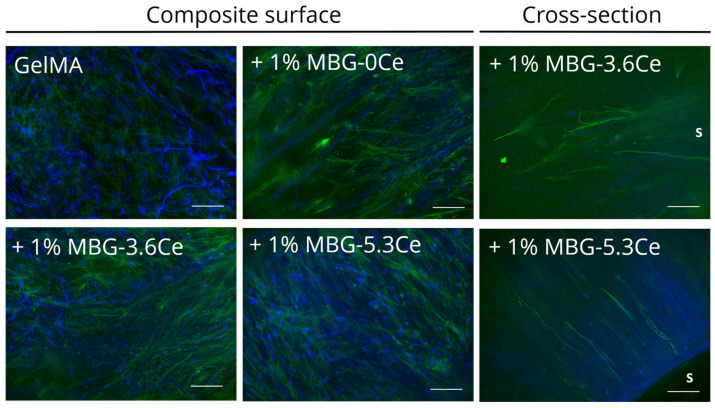
Fluorescence microscopic images of hBMSCs on composites after 28 of culture. In the presented Z-stacks, the cytoskeleton is shown in green, and the nuclei in blue. Scale bar: 100 μm. An “s” indicates the direction towards the surface of the composites.

After 28 days in culture, dense cell layers were present on the composites’ surface irrespective of the type of sample (Figure 6). The scaffolds can be appreciated due to their auto-fluorescing in blue, which has to be distinguished from round blue nuclei. Even though some cells were detectable in the cross-sections orienting along the pores, the majority of cells was present on the materials’ surface.

#### 2.1.5. Release and Uptake of Ions from Composites During Cell Culture

**Figure 7:**
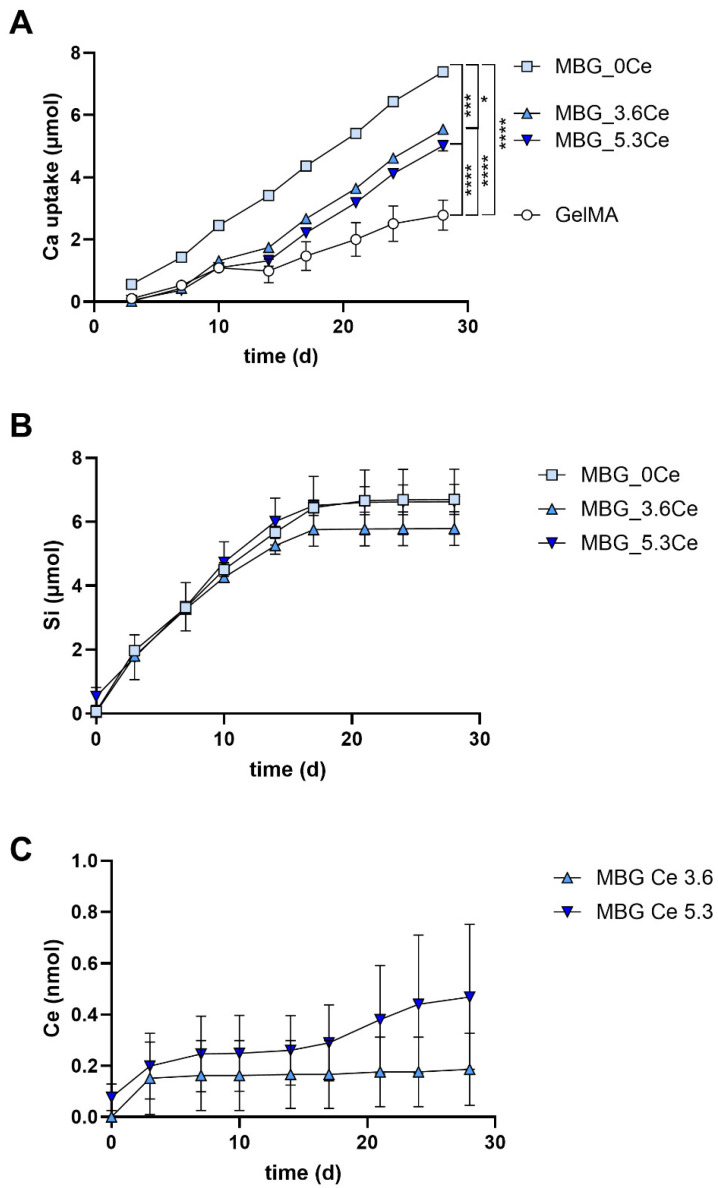
Calcium uptake and silicon/cerium release profiles of composites during cell culture of hBMSCs. Accumulated total uptake of calcium (**A**) and accumulated total release of silicon (**B**) and cerium (**C**) were determined by ICP-OES measurements from cell culture supernatants of composites containing 1% *w*/*v* of different MBGs in comparison to pure GelMA; *n* = 4. Two-way ANOVA, followed by Tukey’s multiple comparison test: * *p* < 0.05; *** *p* < 0.001; **** *p* < 0.0001.

**Figure A9:**
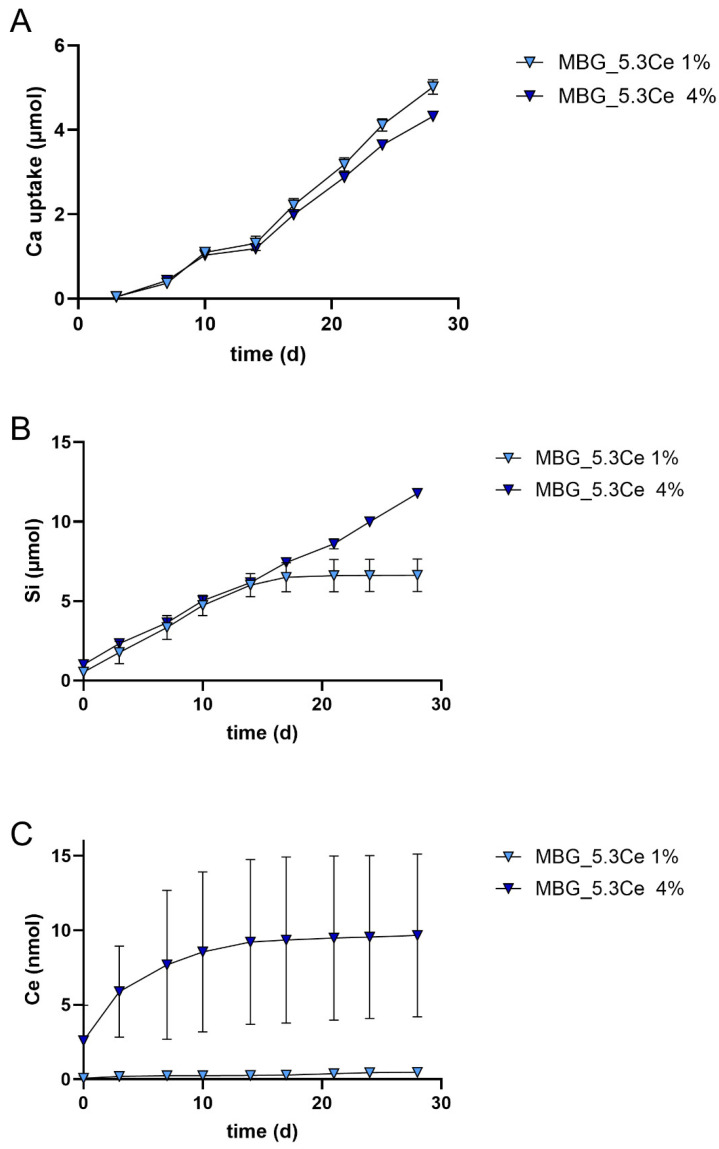
Calcium uptake and silicon/cerium release profiles of composites with 1% and 4% MBG-5.3Ce during cell culture of hBMSCs. Accumulated total uptake of calcium (**A**) and accumulated total release of silicon (**B**) and cerium (**C**) were determined by ICP-OES measurements from cell culture supernatants of composites containing 1% *w*/*v* compared to 4% *w*/*v* of MBG-5.3Ce; *n* = 4.

All composites displayed an uptake of calcium ions from the cell culture medium (Figure 7A and Figure A9A), while silicon ions were released from all MBG-containing composites (Figure 7B and Figure A9B). Given the fact that the medium was changed every three to four days, GelMA + MBG-0Ce took up the highest amount of calcium from the medium, while GelMA demonstrated the lowest calcium uptake during the whole span of the experiment (Figure 7A). The absolute uptake of calcium was comparable with the calcium uptake found in the absence of cells even though slightly lower (Figure 4B). For pure GelMA, there was a higher uptake of calcium in the absence of cells.

Silicon was continuously released from all MBG-containing composites until d17 of cultivation (Figure 7B). During the following days, there were almost no silicon ions detectable for all composites alike, even though only 60% to 75% of originally present silicon ions were released. This might be explained by the growing calcium and cerium phosphate deposits, possibly hindering the release of residual silicon ions. In contrast, when higher amounts of MBG-5.3Ce were used in the constructs, ion release did not stop during the whole incubation period (Figure A9B). Overall, it seems that the presence of cerium in MBGs resulted in a slightly but not significantly altered silicon release profile, e.g., with marginally elevated silicon ion release from GelMA + MBG-5.3Ce on day 10 and 13, while the release was reduced from day 10 onwards for GelMA + MBG-3.6Ce (Figure 7B).

Finally, ICP-OES analysis revealed that GelMA + MBG-3.6Ce and GelMA + MBG-5.3Ce composites released about 0.19 ± 0.14 nmol and 0.47 ± 0.28 nmol of cerium during 28 days of incubation in CCM with hBMSCs (Figure 7C). Of note, the derived concentrations were close to the detection limit of the IPC-OES instrument and thus can only be considered as approximations. As expected, the released amount was higher in the presence of MBG-5.3Ce than with MBG-3.6Ce. Cerium release in both cases was highest after three days in culture, while only for GelMA + MBG-5.3Ce was there a continued slight release until day 28. When higher amounts of MBG-5.3Ce were used in the constructs, cerium release amounted to about 10 nmol during 28 days of incubation in CCM (Figure A9C).

**Figure A10:**
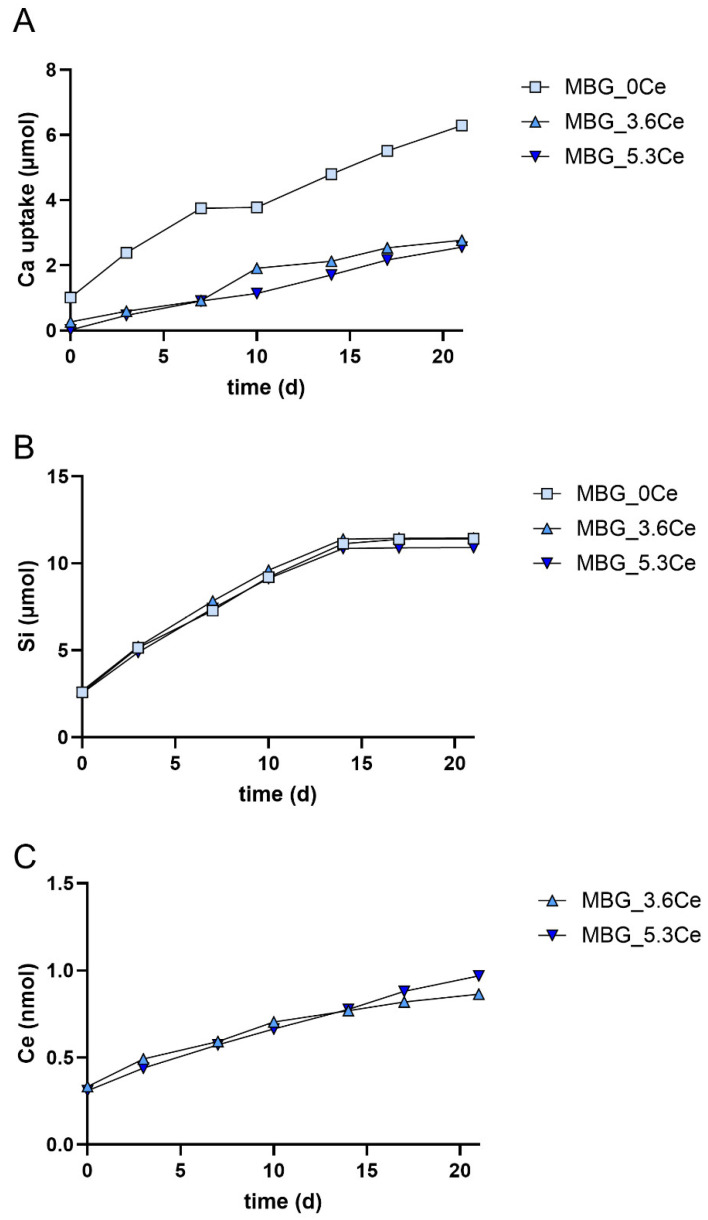
Calcium uptake and silicon/cerium release profiles from pure MBGs during cell culture of hBMSCs. Accumulated total uptake of calcium (**A**) and accumulated total release of silicon (**B**) and cerium (**C**) were determined by ICP-OES measurements in extracts from 1 mg/mL of different MBGs; *n* = 1.

**Figure A11:**
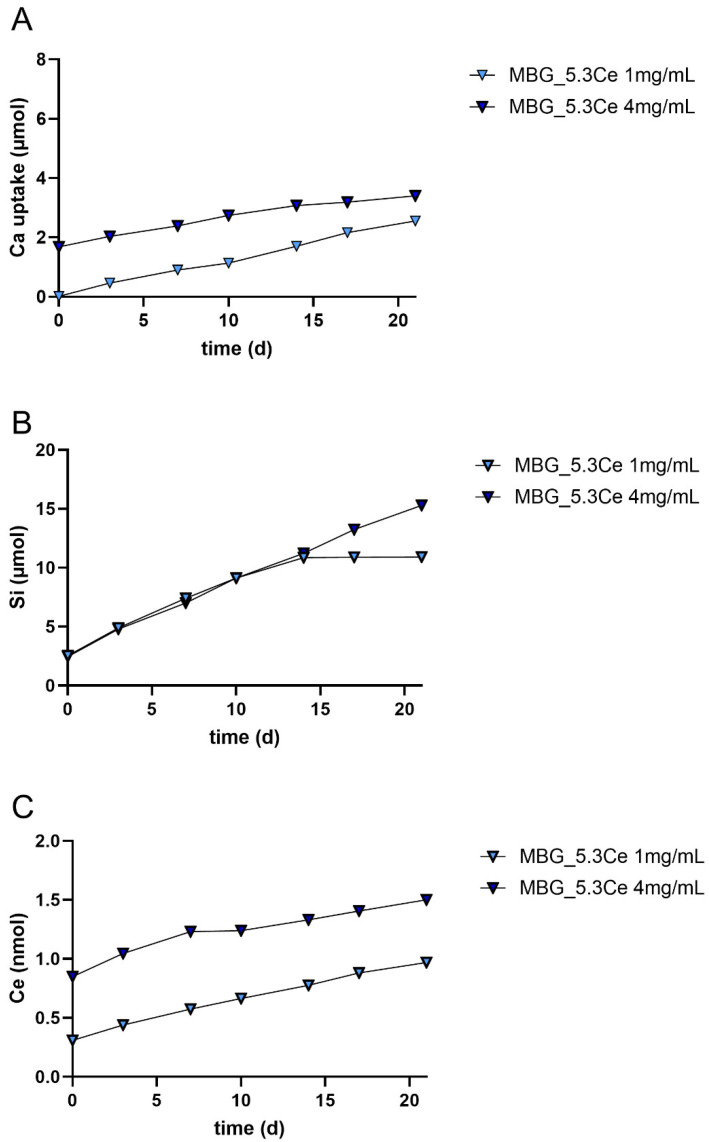
Calcium uptake and silicon/cerium release profiles from pure MBG-5.3Ce during cell culture of hBMSCs. Accumulated total uptake of calcium (**A**) and accumulated total release of silicon (**B**) and cerium (**C**) were determined by ICP-OES measurements in extracts from 1 mg/mL or 4 mg/mL MBG-5.3Ce; *n* = 1.

When comparing calcium uptake and silicon/cerium release profiles of pure MBGs, the latter also displayed an uptake of calcium ions from the cell culture medium used to derive extracts (Figure A10A and Figure A11A), while silicon ions were released from all MBGs (Figure A10B and Figure A11B). Also, here, MBG-0Ce took up the highest amount of calcium from the medium, while Ce-MBG demonstrated markedly lower bioactivity (Figure A10A). Of note, calcium uptake of pure MBGs was lower than that of composites with the same MBG concentrations. In contrast, the silicon ion release was about two-fold higher from bare MBGs at all time points than from composite-bound ones (Figure A10B and Figure A11B). Likewise, the cerium release was about two- to four-fold higher from the former (Figure A10C) and depending on Ce-MBG concentration (Figure A11C).

### 2.2. Discussion

Artificial bone grafts substitutes made of biodegradable polymers and BG nano- and microparticles are considered as promising alternatives for current bone replacement materials [3,6,19]. To date, the majority of research published on biopolymer/BG hybrid materials is on BGN, for which distribution in the polymeric scaffold is not complicated and usually achieved by simple mixing of the components at slightly elevated temperatures with further crosslinking [6,19]. A homogenous distribution of larger particles with a size of tens of microns is considered to be challenging due to their tendency for precipitation and thereby poor dispersibility [24,25].

Thus, there seems to be a limited number of studies demonstrating homogeneous distribution of micro-sized MBGs. Akhtar et al., for instance, claimed to avoid precipitation of 45S5BG microparticles by gradually cooling down GelMA/BG solution under shaking, followed by storage at 4 °C and final crosslinking under UV light [25]. Actual achievement of homogeneous distribution throughout the composites materials was, however, not shown.

In the present study, a homogeneous distribution of MBG particles in GelMA was first attempted by sequential physical and chemical crosslinking analogously to Zheng et al. 2018 [23]. Here, chemical crosslinking was preceded by a physical crosslinking step via cooling down the GelMA/MBG suspension. This was expected to minimize precipitation of MBGs of at the bottom of composites, which was, however, not the case. Thus, we exchanged the physical crosslinking step by quick freezing at −20 °C and only afterwards chemical crosslinking via UV light.

SEM analysis of freeze-dried samples revealed a highly porous structure with elongated shape of the pores, which were additionally oriented from top to bottom of the gels. The latter might be attributed to the quick-freezing step. In contrast, GelMA-based crosslinked hydrogels synthesized in previous work displayed spherically shaped pores [23,26]. Interestingly, there was no severe effect of MBGs on pore sizes. Only for the long axis of the elongated pores did GelMA + MBG-3.6Ce 1% composites display a significant difference to GelMA. Further, at the highest MBG concentration applied, GelMA + MBG-0Ce 4% displayed a slightly changed pore appearance to irregular spherical. In contrast, Mojasteran et al. observed a reduction in pore size when including BGN and Ce-BGN in alginate/gelatin scaffolds [21]. The same reducing effect on pore size was found in a study that included ferric iron/shikonin nanoparticles in a hydrogel made of nitrobenzyl-modified hyaluronic acid and methacrylated silk fibroin [27]. In both studies there was no explanation given for this effect. It might be suggested that an inter-molecular physical/chemical interaction of nanoparticles with the hydrogel components is the reason, which is probably more prominent for the large surface/volume ratio of nanoparticles than for MBGs.

Silicon mapping via EDX analysis indicated that MBGs are rather homogeneously distributed throughout the volume of composites irrespective of MBG-0Ce concentration or cerium content.

Swelling equilibrium was reached after 90–120 min of incubation, while water content and swelling ratio decreased with increasing MBG-0Ce content. This suggests that the higher the MBG content, the less water can be adsorbed by the composites, reflecting a reduced efficacy of body fluid adsorption. In contrast, there is no significant additional impact of cerium on the swelling properties of composites containing 1% MBG. Also, no major difference in swelling in PBS or CCM could be detected.

Akhtar et al. and Ai et al. alike found that increasing content of 45S5BG microparticles in GelMA composites decreased swelling ratio in PBS, even though the particles size of 4 µm is significantly smaller than in the present study [25,28]. As a possible reason for the reduced water absorption properties, it was suggested that BG-derived Na^+^ and Ca^2+^ cations bind to the carboxylic groups of GelMA, while silicon hydroxyl groups bind to amine groups, and thus reduce the number of hydrophilic groups [25]. The swelling equilibrium was, however, reached at 15–30 min and therefore much earlier than in the present study for a comparable composition (5% GelMA, 1–5 wt % BG), while the final swelling ratio in the present study is higher [25]. Following the aforementioned argument, this difference could be related to the probably higher number of cations released from 4 µm 45S5BG. This might be also a result of a presumably different pore geometry due to the different preparation techniques (cooling versus quick freezing). Since there was no analysis of pore microstructure reported, this aspect cannot be directly compared. Further, for composites made of alginate, gelatin, and Ce-BGNs, a reduced swelling ratio could be found compared to pure alginate/gelatin gels, when incubated at 37 °C in PBS for up to 120 h [21].

The Young’s modulus of GelMA and all composite samples were between 19 and 26 kPa. No major differences could be detected for pure GelMA and MBG-containing composites irrespective of the medium (PBS or CCM) used in this study.

In contrast, Akhtar et al. observed higher compressive stiffness between 45 and 60 kPa (5% *w*/*v* GelMA, 1–5% *w*/*v* BG) but with significantly decreased values in the presence of increasing 45S5BG concentrations [25]. They suggested that this was likely related to precipitation of BG particles and thus agglomeration and hindered crosslinking due to increased light reflection. However, agglomeration was rather limited in the present study. And if light was reflected by the present MBGs, this was probably outweighed by improved interfacial bonding and reinforcement. Nevertheless, the mechanical reinforcement aimed for was not achieved. Zheng et al. even reported a detrimental effect of 2.5 to 10% (*w*/*v*) BGN on GelMA/BGN composites and related this to increased light reflection [23]. In contrast, for alginate/gelatin/5% *w*/*v* Ce-BGN composites, a significantly increased compressive modulus was reported [21]. Here, with rather comparable gelatin and BGN amounts (each 5% *w*/*v*), the compressive modulus of the hybrid gel alginate/gelatin was already 13 MPa, which increased to 32 MPa and even 91 MPa for BGN or Ce-BGN-containing composites. Importantly, these materials were double crosslinked by calcium chloride and glutaraldehyde, which, in addition to the 2% (*w*/*v*) alginate, might account for the markedly higher compressive moduli of the pure hybrid gel. Obviously, the use of BGN led to further strongly improved interfacial bonding and reinforcement, which cannot be achieved by BG microparticles. This is in line with a study reporting on a more significant enhancing effect of nanoparticles on the mechanical properties of poly(3hydroxybutyrate)/BG composite systems in comparison to microparticles [24].

In summary, the here observed missing effect of MBGs on composites’ E-modulus might be related to concentration-, size- and chemistry-dependent opposing effects of increased UV light reflection and MBG-related interfacial bonding and reinforcement.

Measuring stability in PBS over 7 days—an important factor for replacement by newly formed bone—revealed rather minimal mass loss of about 10% when comparing composites with increasing MBG-0Ce content with pure GelMA, with the shape of the composites not dramatically altered. This suggests sufficient stability for an about 8 weeks bone repair process, while potentially facilitating new bone formation. Still, stability studies over extended time periods are warranted to confirm this.

In contrast, Akhtar et al. observed a strongly accelerated degradation rate of GelMA-based composites in DMEM when increasing the 45S5 BG concentration to 3% and 5% *w*/*v*, which was suggest to be related to hindered crosslinking, as mentioned above [25]. Here, for a composite with 5% *w*/*v*, the mass loss was about 50 to 60% after 7 days. Since crosslinking density and degradation rate are reported to be inversely proportional [29], this suggests a stronger crosslinking of the present composites than those reported by the named authors. However, the degradation rate depends on many other factors, including composition, particle size, and shape, that are certainly different in both studies.

Mostajeran et al., however, observed a reduced composite degradation at 37 °C in PBS when Ce-BGN was present (mass loss < 10% over 7 days in contrast to a 40% for pure alginate/collagen-scaffolds) [21]. They suggested a neutralizing effect of alkaline ions (e.g., Ca^2+^, Ce^3+^) on acidic byproducts of polymer degradation. However, this might as well be related to the also reported reduced pore size and porosity, restricting water access.

Different to PBS, in CCM, there was a mass increase after 28 days for all composites investigated with no significant differences for non-irradiated samples but with GelMA-+MBG-0Ce displaying the highest value. The latter was even significant after gamma irradiation treatment in comparison to Ce-MBG-containing composites. The absolute mass increase in CCM suggests the formation of precipitates, depending on MBG composition, due to direct contact with CCM, which might include calcium phosphate deposition.

To further elaborate on this aspect and thus to assess the composites’ tendency to integrate with bone, the calcium deposition was analyzed after 28 days in CCM. The strongest calcium deposition was found for GelMA + MBG-0Ce, followed by GelMA + MBG-5.3Ce and GelMA + MBG-3.6Ce. GelMA displayed significantly lower bioactivity than all MBG-containing composites. These results were supported by ICP-OES measurements and visualized by SEM analysis. A reduction in bioactivity of MBGs was also previously reported for melt-quenched as well as sol–gel-derived Ce-MBGs with increasing CeO_2_ [14]. This was also the case when the latter were complexed in alginate beads [14]. This was explained with a lower amount of phosphate groups available for hydroxyapatite layer formation, after binding with cerium ions and forming CePO_4_.

Interestingly, Varini et al. found lower bioactivity of MBGs when incorporated in alginate beads and suggested that alginate inhibits HAP formation by interfering and delaying ionic exchange between MBGs and SBF [14]. Still, in both cases, pure or alginate-bound MBG-0Ce displayed the highest bioactivity. In contrast, we observed slightly lower bioactivity for pure MBGs than those incorporated in GelMA hydrogels when investigating extract and composite supernatants via ICP-OES. This is likely attributed to the additional bioactivity of GelMA. However, ion determination from extracts of pure MBGs were only *n* = 1 and thus need to be further consolidated.

When investigating the cellular response to composites by directly seeding hBMSCs, MBGs decreased cellular attachment and thus seeding efficacy in comparison with pure GelMA. A slight reduction in number of MC3T3-E1 cells after one day of culture was also observed for MBG-containing alginate beads in direct and indirect culture, irrespective of the cerium content [14]. In contrast, Akhtar et al. 2024 reported no significantly altered cellular attachment of human osteoblasts (OBs) on 45S5BG-containing GelMA hydrogels in comparison to pure GelMA [25]. Since Varini et al. reported no cytotoxic behavior of MBG-containing beads to pre-osteoblastic cells MC3T3-C1 cells [14], we suggest that it is rather the altered surface roughness or MBG-induced hydrophobicity that led to reduced initial cell attachment on MBG-containing composites.

Further, the presence of MBGs in the composite decreased proliferation of hBMSCs in comparison to GelMA. This is opposed to findings by Varini et al. [14], who observed an increased proliferation of MC3T3-E1 cells in Ce-containing MBG/alginate beads compared to the control. However, this was only investigated within a short time frame (day 1 to day 4) and only significant in an indirect approach, where cells were not in direct contact with MBGs. This effect was minimized and non-significant when cells were grown in direct contact. In contrast, Akhtar et al. 2024 reported no significantly altered cellular proliferation of human OBs directly grown on 45S5BG-containing GelMA hydrogels in comparison to pure GelMA [25].

The decreased proliferation found here in direct contact is in line with previous findings where cells were found to be less tolerant to BG in case of direct vs. indirect physical interaction [30]. This is also consistent with our own observations on ion dissolution products (IDPs) of bare MBG-3.6Ce and MBG-5.3Ce increasing hBMSC proliferation in contrast to Ce-free MBG-0Ce. Even though cell culture results from extracts were only derived from *n* = 2 and should not be over interpreted, they add valuable information. Here, it has to be emphasized that the MBG concentrations in our direct and indirect culture were the same (e.g., 750 µg MBGs per GelMA/MBG 1% *w*/*v* composite/750 µL medium versus 1 mg/mL bare MBGs), enabling a direct comparison.

Considering that there were higher concentrations of free silicon, calcium, and cerium ions available from extracts of pure Ce-MBGs than from cell culture supernatants of GelMA-bound ones, the lower amount of these cell-instructive IDPs in the latter might be a reasonable explanation for the retarded proliferation on composite materials.

A concentration-dependent stimulatory effect of silicon and cerium ions was reported for the proliferation of MSCs and OBs [31,32,33]. Of note, for cerium, this was found even for concentrations down to 1 nmol/L [31]. While the silicon release from 1 mg/mL bare MBGs was rather comparable, cerium ions were released from 1 mg/mL Ce-MBGs at amounts of about 1.2 µmol/L (~0.9 nmol/750 µL) in total. In addition, there was lower calcium uptake from Ce-MBGs. While high concentrations (>10 mM) of calcium ions are considered cytotoxic to cells [34], lower calcium concentrations have been implicated in retarding proliferation of hBMSCs [35,36]. Thus, the increased hBMSC proliferation in extracts of Ce-MBGs might be related to sufficient cerium release as well as increased concentrations of free calcium ions. A higher cerium release, in addition to a prolonged silicon release, might also be the reason for the slightly increased proliferation of cells on MBG-5.3 4% *w*/*v*-containing composites compared to those with 1% *w*/*v* on days 14/21.

Previous in vitro cell culture studies demonstrated the positive impact of calcium ions on matrix mineralization [37] and silicon ions on the expression of typical osteoblast markers like ALP, osteocalcin, and bone sialoprotein [32,33,38]. Further, improved osteogenic differentiation of hBMSCs was demonstrated on alginate/gelatine/BGN compared to alginate/gelatin, which was further enhanced by Ce-BGNs [21].

In the present study, the presence of Ce-MBGs in composites also resulted in higher specific ALP activities on day 28 compared to GelMA, which was significant for GelMA + MBG-5.3Ce. This suggests a stimulative effect of Ce-MBGs on osteogenic differentiation of hBMSCs. Of note, as in Mostajeran et al. [21], this was achieved without adding dexamethasone. Interestingly also here, ALP activity stayed on a high level starting from day 14 to day 21. Unfortunately, the underlying reasons for the cerium-related effects were not elucidated by the named authors, i.e., via the release of IDPs.

The cerium release form GelMA + MBG-3.6Ce and GelMA + MBG-5.3Ce composites in the present study was about 0.25 µmol/L to 0.63 µmol/L, respectively, and thus two to four times lower than from the corresponding bare MBGs. Both composites showed in addition a reduced calcium uptake compared to GelMA + MBG-0Ce. Further, GelMA-MBG-5.3Ce displayed a slightly higher silicon ion release on days 10/13. Thus, it might be the sum of all these effects observed for the ion release profiles that led to the increase in ALP activity for Ce-containing composites. In line with our observation on composites containing MBG-0Ce and bare MBG-0Ce as being non-osteogenic, Akhtar et al. 2024 also reported no significantly altered ALP activity of human OBs on 45S5BG-containing GelMA hydrogels in comparison to pure GelMA [25].

Interestingly, the stimulating effect on ALP activity was also found for bare Ce-MBGs at 1 mg/mL but was diminished when increasing the concentration to 4 mg/mL for MBG-5.3Ce. For the latter, the calcium uptake was slightly higher, while 1.5 times more cerium ions were released in total. Also, for composites with GelMA + MBG-5.3Ce 4% there was no increase in ALP activity observed compared to those with 1%. Here, cerium ion release was strongly increased but did not result in enhanced osteogenic differentiation. Obviously, higher concentrations of cerium ions have a rather detrimental effect on the osteogenic differentiation of hBMSCs. A concentration-dependent effect of cerium ions was previously described by Zhang et al. for the osteogenic differentiation of primary mouse osteoblasts [31]. Depending on culture time, lower cerium ion concentrations were found to promote differentiation, while being inhibitory at concentrations above 1 µmol/L.

Migration of cells into hydrogel-based composite materials is not often in the focus of research. In the present study, we found a rather limited migration of cells inside the composites after 28 days. Cells rather formed a dense cell layer on the outside. Akhtar et al. 2024 as well reported a dense cell layer of OBs after 21 days on 45S5BG-containing GelMA hydrogels and pure GelMA alike [25]. This is surprising since the pores of the freeze-dried scaffolds seem to be big enough for cells to squeeze in, in particular since these materials are biodegradable. However, Franco et al. reported that pore size measurements with freeze-dried GelMA hydrogels based on SEM might be misleading and not representative for the actual pore size in hydrated condition [39]. In fact, these authors found a significant reduction in pore size for 10% GelMA hydrogels upon rehydration compared to the freeze-dried state, while there was no such discrepancy for alginate-based hydrogels. An in-depth comparison of pore sizes in freeze-dried and hydrated composites would be an interesting task for future investigations. Of note, this effect might be mitigated by choosing biopolymer hybrids such as GelMA/alginate or GelMA/HAMA better supporting cell migration. Another option would be the implementation of GelMA/dextran aqueous two-phase systems for creating interconnected pores [40]. This aspect should be further investigated, including higher initial cell seeding numbers.

In summary, the present study offers improvements and novel insights relative to existing publications in similar domains: Firstly, the here-established composite preparation by rapid freezing and subsequent crosslinking coped with a recognized challenge of providing a rather homogenous distribution of microparticles, which was also demonstrated by EDX mapping. Secondly, GelMA/Ce-MBG provides a more suitable microenvironment for osteogenic differentiation of hBMSCs than those of previously reported alginate/Ce-MBGs. Finally, the direct comparison of ion release and cellular effects of bare MBGs versus composite bound ones offers novel insights on deviating cellular results related to both MBG application forms.

However, we are aware of the potential limitations of our study, which are that cell culture was carried out with hBMSCs of only one donor and in static condition. Analysis with additional donors should be conducted in the future to verify our results accounting for donor variability. Also, hBMSC cultivation on composites might me more beneficial when using perfusion instead of static culture, e.g., with respect to deeper cell infiltration. Further, only ALP and calcium deposition was analyzed for osteogenic differentiation, and the expression of osteogenic genes should be included in future studies of this material.

As a perspective for future investigations, Ce-MBG-containing composites could be exploited as in situ drug delivery systems as was proposed earlier for Ce-MBGs [16,41]. This is since MBGs have high pore volume and specific surface area and a highly ordered structure, allowing for efficient loading and more controlled release kinetics in comparison to melt-derived or traditional sol–gel BG [41].

The antioxidant activity of Ce-MBGs was further improved by loading with different antioxidant molecules like gallic acid, polyphenols, and anthocyanins [16]. Unloaded Ce-MBGs exhibit intrinsic cerium-dependent catalase-like activity, leading to the dismutation of hydrogen peroxide. However, they are only marginally able to dismutate the superoxide anion. This was, however, conveyed via the loading of the named biomolecules giving complementary antioxidant properties. This new property could greatly add to the already promising antioxidant profile of Ce-MBGs, minimizing the total healing time of implants by reducing post-implantation oxidative stress.

In the study by Fraulini et al., MBGs were loaded with gentamycin, a wide-spectrum antibiotic commonly used against bacteria, which cause postoperative infections [41]. Authors could show that an antibacterial effect was present for 10 days of controlled release while MBGs retained bioactivity and antioxidant properties. Thus, Gen-loaded Ce-MBGs were considered to be promising for simultaneous bone regeneration and in situ antibiotic release to tackle one of the most frequent postoperative complications after placing a biomaterial implant.

## 3. Conclusions

The aim of the present study was to create novel composites based on GelMA and Ce-MBG particles and to derive a suitable cellular microenvironment with favorable physicochemical properties, promoting growth and osteogenic differentiation in hBMSCs. Results showed that highly porous composites could be created with MBGs homogeneously distributed throughout the scaffold irrespective of concentration and cerium content. While there were no major differences of the mechanical properties for different MBG compositions and concentrations, the swelling properties decreased with increasing MBG amount. Cerium doping had a significant diminishing impact on bioactivity. This higher availability of calcium ions in the presence of bare and composite-bound Ce-MBG as well as cerium release below 1 µmol/L and a slightly altered release profile of silicon ions for composite-bound Ce-MBG could be responsible for the increased osteogenic differentiation of hBMSCs on cerium-doped composites. These findings thus provide valuable insights on the effects of GelMA/Ce-MBG composites on more physiologically relevant hBMSCs. Future studies should investigate further MBG concentrations with several cell donors, also considering in vivo studies. Additionally, due to the internal porosity of MBGs, these composites should be extended to drug delivery applications by soaking in concentrated drug-containing solutions, further promoting bone cell responses and thus the applicability for bone regeneration and repair.

## 4. Materials and Methods

### 4.1. Materials

GelMA was purchased from Advanced BioMatrix Carlsbad, CA, USA, while MBGs were prepared by evaporation-induced self-assembly process (EISA) as previously described [14]. The molar compositions in mol % of the MBGs used in this study are displayed in Table 1.

Lithium-Phenyl-2,4,6-trimethylbenzoylphosphinate (LAP) was obtained from TCI Deutschland GmbH (Eschborn, Germany). Phosphate-buffered saline (PBS), sterile water, ascorbic acid-2-phosphate, and β-glycerophosphate were obtained from Sigma-Aldrich (Steinheim, Germany). α-MEM, penicillin, and streptomycin were obtained from Merck, Berlin, Germany. iFluor488 phalloidin was purchased from Abcam (Cambridge, UK) and DAPI (4′,6-diamidin-2-phenylindol) from ThermoFisher (Darmstadt, Germany).

### 4.2. Preparation of Composites

The following composite hydrogels were created based on 5% *w*/*v* GelMA: (1) pure GelMA, (2) GelMA + 0.4% *w*/*v* MBG-0Ce, (3) GelMA + 1% *w*/*v* MBG-0Ce, (4) GelMA + 4% *w*/*v* MBG-0Ce, (5) GelMA + 1% *w*/*v* MBG-3.6Ce, and (6) GelMA + 1% *w*/*v* MBG-5.3Ce. Prior to composite hydrogel preparation, Teflon molds (diameter 6.8 mm, height 1.9 mm) were ethanol-wiped and incubated at −80 °C for 60 min. Next, 5% *w*/*v* GelMA solution was prepared by dissolving 200 mg thereof in 4 mL sterile water by constant stirring at 55 °C for 60 min. After a homogeneous solution was obtained, temperature was dropped to 40 °C and aliquots of 900 µL were added to individual vials, containing either 3.96 mg (0.4% *w*/*v*), 9.92 mg (1% *w*/*v*), or 39.6 mg (4% *w*/*v*) MBGs, needed for their different final concentrations within gels, followed by additional stirring for 30 min. Then, 1% *w*/*v* LAP photoinitiator solution was prepared by dissolving 4.5 mg of LAP in 450 µL of deionized water. After that, 90 µL of LAP solution was added to the previously prepared vials containing GelMA and MBGs under continuous stirring. To achieve an instantaneous freezing, 75 µL of the obtained suspensions were applied into Teflon molds directly in a freezer, respectively, and kept frozen for 30 min. The final MBG content/75 µL scaffolds thus equaled to 300 µg (0.4% *w*/*v*), 750 µg (1% *w*/*v*), and 3 mg (4% *w*/*v*). To achieve chemical crosslinking while keeping their frozen state, they were placed on a frozen steel plate and UV-crosslinked (365 nm, 0.17 W/cm^2^, 10 min) using a Herolab UV crosslinking device. Composites were placed at −20 °C overnight and freeze-dried using a Martin Christ Epsilon 2–4 LSC freeze-drying device. Afterwards, samples were washed in deionized water and frozen overnight (ON) at −20 °C again, followed by an additional round of freeze-drying as described. For cell culture experiments, freeze-dried hydrogels were sterilized via gamma irradiation with 30 kGy (BBF Sterilisationservice Gmbh, Kernen, Germany).

### 4.3. Scanning Electron Microscopy (SEM) and Energy-Dispersive X-Ray Spectroscopy (EDX)

The average size of bare MBGs was analyzed with SEM. To investigate intrinsic morphological properties as well as pore size and MBG distribution within the freshly prepared composites, samples of each type were cut into two halves with a scalpel to obtain cross-sections. Next to these, we also analyzed the outer surfaces of the composites after incubating them in CCM for 28 days to visualize calcium deposits (see Section 4.6). Both sample types were sputter-coated with a carbon layer (Plano, Wetzlar, Germany) and analyzed by scanning electron microscopy (Zeiss DSM 980 Gemini) and energy-dispersive X-ray spectroscopy (EDX) combined with elemental mapping (Zeiss DSM 950, System 7 EDX). Acceleration voltages were 3 and 10 kV for SEM (high-vac mode) and 15 kV (high-vac mode) for EDX, respectively. Average size of MBGs (*n* = 20) and pore sizes in the composite hydrogel (*n* = 100) were calculated via Image J software (version 1.54f). For quantifying the latter, two orthogonal axes (long and short) were identified for each pore.

### 4.4. Swelling Properties

Swelling properties of the hydrogels in phosphate-buffered saline (PBS) or α-MEM supplemented with 10% fetal calf serum (FCS), 100 U/mL penicillin, and 100 µg/mL streptomycin (CCM) in a humidified atmosphere at 37 °C and 5% CO_2_ were determined gravimetrically. Briefly, 1 mL of medium was added to each of the samples and incubated as indicated. At certain intervals, gels were taken out and their mass was measured gravimetrically [23] using a five-decimal analytical balance (Sartorius, Göttingen, Germany). Swelling ratio (*SR*) and water content (*WC*) were analyzed as previously described [42,43] and according to Formulas (1) and (2) [23].

(1) $$SR=\frac{m\left(t\right)-m\left(d\right)}{m\left(d\right)}$$

(2) $$WC=\frac{m\left(t\right)-m\left(d\right)}{m\left(t\right)}\times 100\%$$

Here, *m*(*d*) and *m*(*t*) are sample mass before (freeze-dried) and after a particular swelling time. The sampling size of four replicas was considered sufficient for each sample type.

### 4.5. Mechanical Characterization

The mechanical properties of the swollen composites were analyzed with a Microsquisher^®^ (CellScale, Waterloo, ON, Canada) according to Al-Maawi et al. [43]. In brief, the composites were swollen for 1 h in either PBS or CCM in a humidified atmosphere at 37 °C and 5% CO_2_. Next, cylinders with a diameter of 3 mm were punched from each gel with a biopsy punch and their force–displacement curves were analyzed using 50% displacement during a loading phase of 2 min in a 37 °C temperature bath of the medium. The compressive modulus was calculated as the slope of the initial linear region (0–15%) of the strain–stress curves. Three replicas were investigated for each sample type.

### 4.6. Stability and Bioactivity of the Composites in PBS and Cell Culture Media

Composite stability was analyzed gravimetrically using a microbalance with samples incubated either in PBS for 7 days or in CCM for 28 days in a humidified atmosphere at 37 °C and 5% CO_2_. In PBS, composites containing 0.4, 1, or 4% MBG-0Ce were compared to pure GelMA, while in CCM, this was performed for those with 1% MBG-0Ce, -3.6Ce, and -5.3Ce. In order to estimate the effect of gamma irradiation on the composites, two sets of the gels were analyzed in CCM: one without sterilization and one with. The conditions and the time points of the medium change were exactly as in the following cell culture experiments: After 28 days, with medium changes every third to fourth day, the medium was removed and the gels were washed two times with 1 mL of sterile water (each time for 30 min), followed by freeze-drying overnight. Finally, their mass was measured gravimetrically, and the mass change (*MC*) was calculated according to Formula (3):

(3) $$MC=\frac{m\left(a\right)-m\left(0\right)}{m\left(0\right)}\times 100\;\%$$

Here, *m*(0) is the initial mass of the freeze-dried samples before the experiment, and *m*(*a*) is the mass of the freeze-dried samples after 28 days of swelling in medium; *n* = 3.

Next, the non-gamma-irradiated scaffolds were prepared for SEM-analysis as described above (see 4.3) for visualizing calcium deposits (bioactivity), while the gamma-irradiated samples were submitted to quantitative calcium determination (see below).

Calcium depositions over 28 days were quantified by Fluitest^®^ Ca-CPC for composites incubated in CCM supplemented with 5 mM β-glycerophosphate and 50 µM ascorbic acid-2-phosphate (complete CCM) after following the same routine of medium changes every third to fourth day. The named supplements were added to CCM to allow for assessing bioactivity in the later-applied cell culture conditions (see Section 4.7), enabling the comparison of settings with and without cells. In brief, after 28 days of incubation in medium, the latter was removed, and the scaffolds were washed with pre-warmed PBS and frozen at −80 °C. The hydrogels were first mechanically destroyed in 1% Triton X-100 (Sigma-Aldrich)/PBS using two pipet tips and further sonicated for 10 min. This suspension was air-dried, and 0.25 M HCl was added to perform calcium quantification at 590 nm using an automated Tecan plate reader. A calibration curve was also prepared from different concentrations of CaCl_2_ (0–2.5 mmol/L). To allow for a second independent assessment of calcium deposition, calcium withdrawal from the supernatants of each medium change was analyzed by collecting and freezing (−80 °C) them to later perform inductively coupled plasma–optical emission spectrometry (ICP-OES) measurements (see Section 4.8).

### 4.7. Cell Culture Experiments

hBMSCs were generously provided by Prof. Bornhäuser and his team at MedicalClinic I, University Hospital Dresden. These cells were isolated from the bone marrow aspirate of a 25-year-old male donor and subsequently expanded. The hBMSCs were cultured in α-MEM supplemented with 10% fetal calf serum (FCS), 100 U/mL penicillin, and 100 µg/mL streptomycin (CCM), maintained in a humidified atmosphere at 37 °C with 5% CO_2_.

#### 4.7.1. Indirect Cell Culture

The experiment was conducted using the adapted protocol from Westhauser et al. [17], exposing hBMSCs to IDPs derived from MBG extracts under sterile conditions. To derive extracts, 15 mg of MBG-0Ce, MBG-3.6Ce, and MBG-5.3Ce and 60 mg of MBG-5.3Ce were incubated in 15 mL CCM in a humidified atmosphere at 37 °C and 5% CO_2_ (1 mg/mL or 4 mg/mL in the final suspension). After 3 days in CCM, the conditioned CCM was removed, centrifuged, filtered with 0.22 µm syringe filters (Sigma-Aldrich), and collected. Then, β-glycerophosphate and ascorbic acid-2-phosphate were added to the extract to reach the final concentration of 5 mM and 50 µM, respectively. A 500 µL sample of each derived extract was removed and frozen for later assessment of calcium, silicon, and cerium ions via IPC-OES (see Section 4.8). Then, 15 mL of fresh CCM was added to the MBGs for continued extract generation. In the following, CCM was removed and replaced as indicated after an additional 3, 7, 10, 14, and 21 days.

In parallel, hBMSCs were seeded at 10,000 cells/cm^2^ in 48-well tissue culture plates (*n* = 2) in 750 µL MBG extracts/well derived after the first three days of conditioning in CCM and supplemented with β-glycerophosphate and ascorbic acid-2-phosphate. Additionally, a control group was seeded in conditioned pure CCM (no MBGs) containing β-glycerophosphate and ascorbic acid-2-phosphate (complete CCM). Media changes were conducted twice weekly using the conditioned CCM after day 3, 7, 10, 14, and 21 of extract generation (or pure CCM in case of the control group) as described above. After 1, 7, 14, and 23 days of cultivation, the cells were harvested by removing media and washing with pre-warmed PBS, followed by freezing at −80 °C. They were then subjected to analysis for proliferation and osteogenic differentiation.

#### 4.7.2. Direct Cell Culture

In preparation for cell seeding, the gamma-sterilized samples GelMA, GelMA + 1%MBG-0Ce, GelMA + 1%MBG-3.6Ce, GelMA + 1%MBG-5.3Ce, and GelMA + 4%MBG-5.3Ce (*n* = 4; V = 75 µL) were placed directly at the bottom of in-house-made Teflon rings located in 48-well plates. After that, 150 µL of pre-warmed complete CCM (37 °C) was applied to each scaffold in the rings and incubated for 30 min in the cell incubator at 37 °C, 5% CO_2_, followed by the addition of 200 µL of complete CCM and swelling for 24 h in the cell incubator. A quantity of 20,000 hBMSCs per 100 µL complete CCM was then added to the top of the rings to let the cells attach to the scaffolds during the next 24 h in the cell incubator. Next day, the composites were harvested for time point day 1 after washing two times with pre-warmed PBS and frozen. Remaining scaffolds were gently transferred from the Teflon rings to fresh 48-well plates with the addition of 300 µL of fresh complete CCM (total volume 750 µL to simulate the same theoretical concentration of MBGs in the composites as in indirect cell culture (1 mg/mL and 4 mg/mL MBGs). The medium was further changed twice a week. Before each harvesting day, the supernatants were collected to determine ion profile release profiles via ICP-OES. Additionally, a plate where only scaffolds were present without cells was conditioned until day 28. After 1, 7, 14, and 23 days of cultivation, the composites were harvested by removing media and washing with pre-warmed PBS, followed by freezing at −80 °C for subsequent analysis of proliferation and osteogenic differentiation.

### 4.8. Ion Release Profiles by ICP-OES

The calcium, silica, and cerium ion concentrations in supernatants derived at each medium change were determined with ICP-OES, (Plasmaquant Elite, Analytic Jena, Jena, Germany) as previously described [44]. Ultrapure water with 2% nitric acid was used for sample preparation and all dilutions. Calibrant solutions for silicon, calcium, and cerium were prepared from certified solutions (TraceCERT Merck Millipore, Darmstadt, Germany). The use of glass equipment was avoided during sample preparation to prevent Si contamination. Disposable polypropylene tubes were used for sample collection and dilution.

### 4.9. Colorimetric Measurements

Proliferation and osteogenic differentiation were assessed by LDH and alkaline phosphatase activity (ALP), respectively [26,44]. All measurements were performed with cell lysates obtained after 1, 7, 14, 21, and 28 days of cultivation using 1% Triton X-100 in PBS. The hydrogels were first mechanically destroyed in the Triton X-100 solution using two pipet tips and sonicated for 10 min. The suspensions were transferred to low-binding tubes and centrifuged at 15,000 rpm for 3 min. Supernatants were then analyzed in duplicate. LDH activity was determined using an LDH cytotoxicity detection kit (Takara, Saint-Germain-en-Laye, France). A 50 µL quantity of the cell lysate was mixed with 50 µL of LDH substrate buffer, and the enzymatic reaction was stopped with 50 µL of 0.5 M HCl after 6 min. Absorbance at 492 nm was measured (Infinite^®^ M200 Pro microplate reader, Tecan Trading AG, Männedorf, Switzerland). Similarly, cell lysates of defined cell numbers were used to prepare a calibration line. ALP activity was determined using p-nitrophenyl phosphate as substrate. A 25 µL aliquot of the cell lysate was added to 125 µL of ALP substrate buffer containing 1 mg/mL p-nitrophenyl phosphate (Sigma-Aldrich), 0.1 M diethanolamine, 1 mM MgCl_2_, and 0.1% Triton X-100 (pH 9.8). The mixture was incubated at 37 °C for 30 min before the enzymatic reaction was stopped by adding 65 µL of 1 M NaOH. Next, the plates were centrifuged at 4000 rpm for 15 min and 170 µL of the supernatants were transferred to fresh plates. Finally, the absorbance was measured at 405 nm (Tecan microplate reader). A calibration curve was prepared from different concentrations of p-nitrophenol. ALP activity was normalized to the cell number determined by LDH quantification.

### 4.10. Immunofluorescence Staining of f-Actin and Nuclei

Composites were fixed overnight in 4% (*v/v*) paraformaldehyde, transferred to 0.4% (*v/v*) paraformaldehyde, and then washed three time with PBS and an orbital shaker. Afterwards, they were cut in halves with a scalpel and permeabilized with 0.2% Triton X100 in PBS for 5 min. Then, a blocking step in 1% (*w*/*v*) BSA in PBS was performed for 30 min. Staining was performed with 1 μL/mL iFluor488 phalloidin and 1 µg/mL DAPI in 1% BSA/PBS for 1 h. After final washing with PBS, samples were imaged using a Keyence BZ-X800 fluorescence microscope, Neu-Isenburg, Germany. Due to the roughness of the sample surface, images were reconstructed from Z-stacks.

### 4.11. Statistics

Statistical analysis was conducted in OriginPro 2018 Software. One-way and two-way analysis of variance (ANOVA) with either Dunn–Sidak or Tukey post hoc test was applied to evaluate differences between groups. *p* values < 0.05 were considered statistically significant. All the experiments were conducted with at least with three replicas if not stated otherwise stated.

## Figures and Tables

**Table 1:** Nominal composition (mol%) of MBGs [14].

Glass	SiO_2_	CaO	P_2_O_5_	CeO_2_
MBG-0Ce	80.0	15.0	5.0	-
MBG-3.6Ce	77.1	14.5	4.8	3.6
MBG-5.3Ce	75.8	14.2	4.7	5.3

## Data Availability

Original microscopy images related to this study are publicly available in Figshare repository at URL: https://doi.org/10.6084/m9.figshare.29197247. All other raw data supporting this study´s findings are available upon reasonable request from the corresponding author.

## Abbreviations

The following abbreviations are used in this manuscript:

BGs	bioactive glasses
BGNs	bioactive glass nanoparticles
Ce-MBGs	cerium-doped mesoporous BG microparticles
CCM	cell culture medium
EDX	energy-dispersive X-ray spectroscopy
ICP-OES	inductively coupled plasma–optical emission spectrometry
IDPs	ion dissolution products
GelMA	methacrylated gelatin
hBMSCs	human mesenchymal stem cells
OBs	osteoblasts
ROS	reactive oxygen species
SEM	scanning electron microscopy

## References

[1] Hak D.J., Fitzpatrick D., Bishop J.A., Marsh J.L., Tilp S., Schnettler R., Simpson H., Alt V. (2014). Delayed union and nonunions: Epidemiology, clinical issues, and financial aspects. Injury.

[2] Wang C., Shen H., Tian Y., Xie Y., Li A., Ji L., Niu Z., Wu D., Qiu D. (2014). Bioactive nanoparticle-gelatin composite scaffold with mechanical performance comparable to cancellous bones. ACS Appl. Mater. Interfaces.

[3] Bai X., Gao M., Syed S., Zhuang J., Xu X., Zhang X.Q. (2018). Bioactive hydrogels for bone regeneration. Bioact. Mater..

[4] Celikkin N., Mastrogiacomo S., Jaroszewicz J., Walboomers X.F., Swieszkowski W. (2018). Gelatin methacrylate scaffold for bone tissue engineering: The influence of polymer concentration. J. Biomed. Mater. Res. A.

[5] Fang X., Xie J., Zhong L., Li J., Rong D., Li X., Ouyang J. (2016). Biomimetic gelatin methacrylamide hydrogel scaffolds for bone tissue engineering. J. Mater. Chem. B.

[6] Sergi R., Bellucci D., Cannillo V. (2020). A Review of Bioactive Glass/Natural Polymer Composites: State of the Art. Materials.

[7] Kokubo T., Kim H.-M., Kawashita M. (2003). Novel bioactive materials with different mechanical properties. Biomaterials.

[8] Hench L.L., Splinter R.J., Allen W.C., Greenlee T.K. (1971). Bonding mechanisms at the interface of ceramic prosthetic materials. J. Biomed. Mater. Res..

[9] Hoppe A., Güldal N.S., Boccaccini A.R. (2011). A review of the biological response to ionic dissolution products from bioactive glasses and glass-ceramics. Biomaterials.

[10] Cacciotti I. (2017). Bivalent cationic ions doped bioactive glasses: The influence of magnesium, zinc, strontium and copper on the physical and biological properties. J. Mater. Sci..

[11] Rahmati M., Mozafari M. (2020). Selective Contribution of Bioactive Glasses to Molecular and Cellular Pathways. ACS Biomater. Sci. Eng..

[12] Kargozar S., Baino F., Hoseini S.J., Hamzehlou S., Darroudi M., Verdi J., Hasanzadeh L., Kim H.-W., Mozafari M. (2018). Biomedical applications of nanoceria: New roles for an old player. Nanomedicine.

[13] Nicolini V., Gambuzzi E., Malavasi G., Menabue L., Menziani M.C., Lusvardi G., Pedone A., Benedetti F., Luches P., D’Addato S. (2015). Evidence of catalase mimetic activity in Ce^3+^/Ce^4+^ doped bioactive glasses. J. Phys. Chem. B.

[14] Varini E., Sánchez-Salcedo S., Malavasi G., Lusvardi G., Vallet-Regí M., Salinas A.J. (2019). Cerium (III) and (IV) containing mesoporous glasses/alginate beads for bone regeneration: Bioactivity, biocompatibility and reactive oxygen species activity. Mater. Sci. Eng. C. Mater. Biol. Appl..

[15] Zambon A., Malavasi G., Pallini A., Fraulini F., Lusvardi G. (2021). Cerium Containing Bioactive Glasses: A Review. ACS Biomater. Sci. Eng..

[16] Lusvardi G., Fraulini F., D’Addato S., Zambon A. (2022). Loading with Biomolecules Modulates the Antioxidant Activity of Cerium-Doped Bioactive Glasses. ACS Biomater. Sci. Eng..

[17] Westhauser F., Rehder F., Decker S., Kunisch E., Moghaddam A., Zheng K., Boccaccini A.R. (2021). Ionic dissolution products of Cerium-doped bioactive glass nanoparticles promote cellular osteogenic differentiation and extracellular matrix formation of human bone marrow derived mesenchymal stromal cells. Biomed. Mater..

[18] Barreto M.E.V., Medeiros R.P., Shearer A., Fook M.V.L., Montazerian M., Mauro J.C. (2022). Gelatin and Bioactive Glass Composites for Tissue Engineering: A Review. J. Funct. Biomater..

[19] Utech S., Boccaccini A.R. (2016). A review of hydrogel-based composites for biomedical applications: Enhancement of hydrogel properties by addition of rigid inorganic fillers. J. Mater. Sci..

[20] Gao C., Gao Q., Li Y., Rahaman M.N., Teramoto A., Abe K. (2013). In vitro evaluation of electrospun gelatin-bioactive glass hybrid scaffolds for bone regeneration. J. Appl. Polym. Sci..

[21] Mostajeran H., Baheiraei N., Bagheri H. (2024). Effects of cerium-doped bioactive glass incorporation on an alginate/gelatin scaffold for bone tissue engineering: In vitro characterizations. Int. J. Biol. Macromol..

[22] Lu B., Zhu D.-Y., Yin J.-H., Xu H., Zhang C.-Q., Ke Q.-F., Gao Y.-S., Guo Y.-P. (2019). Incorporation of cerium oxide in hollow mesoporous bioglass scaffolds for enhanced bone regeneration by activating the ERK signaling pathway. Biofabrication.

[23] Zheng J., Zhao F., Zhang W., Mo Y., Zeng L., Li X., Chen X. (2018). Sequentially-crosslinked biomimetic bioactive glass/gelatin methacryloyl composites hydrogels for bone regeneration. Mater. Sci. Eng. C. Mater. Biol. Appl..

[24] Misra S.K., Mohn D., Brunner T.J., Stark W.J., Philip S.E., Roy I., Salih V., Knowles J.C., Boccaccini A.R. (2008). Comparison of nanoscale and microscale bioactive glass on the properties of P(3HB)/Bioglass composites. Biomaterials.

[25] Akhtar M., Peng P., Bernhardt A., Gelinsky M., Ur Rehman M.A., Boccaccini A.R., Basu B. (2024). Gelatin Methacryloyl (GelMA)—45S5 Bioactive Glass (BG) Composites for Bone Tissue Engineering: 3D Extrusion Printability and Cytocompatibility Assessment Using Human Osteoblasts. ACS Biomater. Sci. Eng..

[26] Heinemann C., Buchner F., Lee P.S., Bernhardt A., Kruppke B., Wiesmann H.-P., Hintze V. (2023). Effects of Gamma Irradiation and Supercritical Carbon Dioxide Sterilization on Methacrylated Gelatin/Hyaluronan Hydrogels. J. Funct. Biomater..

[27] Chen X., Li Z., Ge X., Qi X., Xiang Y., Shi Y., Li Y., Pan Y., Wang Y., Ru Y. (2024). Ferric Iron/Shikonin Nanoparticle-Embedded Hydrogels with Robust Adhesion and Healing Functions for Treating Oral Ulcers in Diabetes. Adv. Sci..

[28] Ai Y., Dai F., Li W., Xu F., Yang H., Wu J., Yang K., Li L., Ai F., Song L. (2023). Photo-crosslinked bioactive BG/BMSCs@GelMA hydrogels for bone-defect repairs. Mater. Today Bio..

[29] Zeimaran E., Pourshahrestani S., Fathi A., Razak N.A.B.A., Kadri N.A., Sheikhi A., Baino F. (2021). Advances in bioactive glass-containing injectable hydrogel biomaterials for tissue regeneration. Acta Biomater..

[30] Qazi T.H., Hafeez S., Schmidt J., Duda G.N., Boccaccini A.R., Lippens E. (2017). Comparison of the effects of 45S5 and 1393 bioactive glass microparticles on hMSC behavior. J. Biomed. Mater. Res. A.

[31] Zhang J., Liu C., Li Y., Sun J., Wang P., Di K., Zhao Y. (2010). Effect of cerium ion on the proliferation, differentiation and mineralization function of primary mouse osteoblasts in vitro. J. Rare Earths.

[32] Han P., Wu C., Xiao Y. (2013). The effect of silicate ions on proliferation, osteogenic differentiation and cell signalling pathways (WNT and SHH) of bone marrow stromal cells. Biomater. Sci..

[33] Costa-Rodrigues J., Reis S., Castro A., Fernandes M.H. (2016). Bone Anabolic Effects of Soluble Si: In Vitro Studies with Human Mesenchymal Stem Cells and CD14+ Osteoclast Precursors. Stem Cells Int..

[34] Maeno S., Niki Y., Matsumoto H., Morioka H., Yatabe T., Funayama A., Toyama Y., Taguchi T., Tanaka J. (2005). The effect of calcium ion concentration on osteoblast viability, proliferation and differentiation in monolayer and 3D culture. Biomaterials.

[35] Mahajan A., Alexander L.S., Seabolt B.S., Catrambone D.E., McClung J.P., Odle J., Pfeiler T.W., Loboa E.G., Stahl C.H. (2011). Dietary calcium restriction affects mesenchymal stem cell activity and bone development in neonatal pigs. J. Nutr..

[36] González-Vázquez A., Planell J.A., Engel E. (2014). Extracellular calcium and CaSR drive osteoinduction in mesenchymal stromal cells. Acta Biomater..

[37] Nakamura S., Matsumoto T., Sasaki J.-I., Egusa H., Lee K.Y., Nakano T., Sohmura T., Nakahira A. (2010). Effect of calcium ion concentrations on osteogenic differentiation and hematopoietic stem cell niche-related protein expression in osteoblasts. Tissue Eng. Part A.

[38] Reffitt D.M., Ogston N., Jugdaohsingh R., Cheung H.F.J., Evans B.A.J., Thompson R.P.H., Powell J.J., Hampson G.N. (2003). Orthosilicic acid stimulates collagen type 1 synthesis and osteoblastic differentiation in human osteoblast-like cells in vitro. Bone.

[39] Franco A., Van Durme B., Van Vlierberghe S., Dupont-Gillain C. (2024). Misleading Pore Size Measurements in Gelatin and Alginate Hydrogels Revealed by Confocal Microscopy. Tissue Eng. Part C. Methods.

[40] Ben Messaoud G., Aveic S., Wachendoerfer M., Fischer H., Richtering W. (2023). 3D Printable Gelatin Methacryloyl (GelMA)-Dextran Aqueous Two-Phase System with Tunable Pores Structure and Size Enables Physiological Behavior of Embedded Cells In Vitro. Small.

[41] Fraulini F., Raimondi S., Candeliere F., Ranieri R., Zambon A., Lusvardi G. (2023). Ce-MBGs Loaded with Gentamicin: Characterization and In Vitro Evaluation. J. Funct. Biomater..

[42] Rother S., Galiazzo V.D., Kilian D., Fiebig K.M., Becher J., Moeller S., Hempel U., Schnabelrauch M., Waltenberger J., Scharnweber D. (2017). Hyaluronan/Collagen Hydrogels with Sulfated Hyaluronan for Improved Repair of Vascularized Tissue Tune the Binding of Proteins and Promote Endothelial Cell Growth. Macromol. Biosci..

[43] Al-Maawi S., Rother S., Halfter N., Fiebig K.M., Moritz J., Moeller S., Schnabelrauch M., Kirkpatrick C.J., Sader R., Wiesmann H.-P. (2022). Covalent linkage of sulfated hyaluronan to the collagen scaffold Mucograft^®^ enhances scaffold stability and reduces proinflammatory macrophage activation in vivo. Bioact. Mater..

[44] Kroschwald L.M., Allerdt F., Bernhardt A., Rother S., Zheng K., Maqsood I., Halfter N., Heinemann C., Möller S., Schnabelrauch M. (2021). Artificial Extracellular Matrices Containing Bioactive Glass Nanoparticles Promote Osteogenic Differentiation in Human Mesenchymal Stem Cells. Int. J. Mol. Sci..

